# Metamaterial properties of Babinet complementary complex structures

**DOI:** 10.1038/s41598-023-31685-7

**Published:** 2023-03-22

**Authors:** Emese Tóth, Balázs Bánhelyi, Olivér Fekete, Mária Csete

**Affiliations:** 1 Department of Optics and Quantum Electronics, University of Szeged, Dóm tér 9, Szeged, 6720 Hungary; 2 Department of Computational Optimization, University of Szeged, Árpád tér 2, Szeged, 6720 Hungary

**Keywords:** Metamaterials, Metamaterials

## Abstract

Single and multiple layers of sub-wavelength periodic Babinet complementary patterns composed of rounded nano-object miniarrays were investigated. In case of illumination with linearly and circularly polarized light the azimuthal orientation and handedness (in)dependence of (cross-polarized) copolarized transmitted signal components was proven for all types of patterns. Considerable (weak) asymmetric transmission was demonstrated in extended bands exclusively for both types of copolarized (cross-polarized) signals transmitted through single layer of convex miniarrays. Three-dimensional structures constructed with convex–concave–convex complex pattern-layers resulted in a negative index at the visible region boundary both for linearly and circularly polarized light illuminations. This is because dipolar modes on the convex nano-objects are synchronized with co-existent reversal dipoles on the concave nano-objects via interlayer coupling. Although during linearly polarized light illumination, the interlayer interaction decouples the localized and propagating modes excitable on the concave pattern in the 90° azimuthal orientation, the synchronization via tilted-rotating nanoring dipoles is almost perfect in the 0° azimuthal orientation. For circularly polarized light illumination, both the dispersion maps and the negative index phenomena synthesize the characteristics of the two orthogonal linearly polarized light illuminations. Important aspect is the appearance of a small/intermediate (large) time-averaged amplitude magnetic dipole due to the tilted (twisted) electric dipole on the concave nanoring, which less/more quickly turns (continuously rotates) with large/intermediate (small) out-of-plane tilting, when illumination is realized with linearly polarized light in the 90°/0° azimuthal orientation (with circularly polarized light). The location of the negative index can be predicted based on the copolarized transmittance signals computed for circularly polarized light illumination by using the linear base representation of Jones transmission matrix elements.

## Introduction

Metamaterials are composed of periodic arrays of subwavelength individual nano-objects or miniarrays of them, which allow to achieve phenomena not possible with the basic constituents, including bianisotropy, reciprocity, chirality, artificial magnetization and boosted Fano-effect^1–4^. The effective optical properties of metamaterials can be determined through retrieval procedures, which allow to identify the spectral regions of negative refraction, as well as to qualify nonreciprocal materials in case of proper optical signal transformation^5–10^.

Various configuration tailoring methods have been developed to tune the spectral properties of metamaterials. The possibility to tune magnetic resonances into the visible spectral region through designed asymmetric rings of plasmonic nanoparticles has been demonstrated^2^. In rectangular arrays of split-ring-resonators (SRRs) supporting magnetic dipoles, the spectral position of the plasmonic resonance remains unaltered, whereas the full-width-at-half-maximum (FWHM) can be tuned by varying the composing antennas’ density and the angle of incidence^11^. To overcome the saturation of the magnetic response around 100 THz, the use of larger number of splits and silver has been proposed^12,13^.

A negative index material (NIM) has been achieved via plasmonic resonance excitation on SRRs in rectangular arrays, by driving dipoles along the capacitive gaps in case of polarization aligned with them^14,15^.

The extension into three-dimensions opened novel possibilities, e.g., mimicking optical magnetism, when magnetic resonances of different orders are excited on U-shaped blocks arranged in an upright orientation in rectangular patterns^1,15,16^. The capability of C-shaped apertures to extend the NIM phenomenon into wide spectral regions has been demonstrated^17^.

For application purposes those metamaterials are particularly promising, which exhibit NIM phenomenon originating from chirality, due to the hybridized plasmon modes that are handedness sensitive^18–23^. Parity-time (PT) phase transition was demonstrated in the polarization response of a C-shaped aperture array with anisotropic absorption^24^. The C-shaped antenna-arrays are promising, because the chirality and PT symmetry can be superimposed at perpendicular incidence, whereas under oblique incidence tuneable asymmetric transmission, optical activity and ellipticity can be achieved^25,26^. The polarization manipulation Pancharatnam-Berry phase effects achievable using in-plane arrays of C-shaped structures can be used in nonlinear phenomena excitation^27^. The beam steering phase effects in layered arrays of C-shaped structures are promising in information encoding^28^.

Several examples prove that various types of single and multilayer complementary patterns are incorporable into compact nanoplasmonic circuitries. The Babinet principle was applied to design artificial metamaterials based on concave split ring resonators (c-SRRs)^29^. It has been demonstrated that the complementary SRRs and c-SRRs exhibit complementary spectral responses as well as a near-field distribution of the respective eigenmodes^30^. The wave-front control of cross-polarized light via Babinet inverted nanoantennas was used to create metalenses^31^. The double NIM phenomenon has been achieved with planar metamaterials using the Babinet principle, where the negative ε and μ originate from the film and the embedded aperture, respectively^32^. The dual band chirality and NIM phenomenon have also been predicted in the near-infrared in the case of bilayer**-**rotated U-shaped complementary apertures^33^. The orientation-dependent bianisotropy of c-SRRs has been used to control the phase, orientation and ellipticity of the cross-polarized evanescent waves (serving as an all-optical analogue of the Smith–Purcell phenomenon related) emission^34,35^. Second-order vortex beams have been generated, via rotation gradient nonlinear metasurfaces made of c-SRRs, with orbital-angular-momentum and diffraction angle determined by the spin-states of both harmonics^36^. Optical activity comparable with three-dimensional (3D) chiral materials and circular polarization-dependent conversion rates has been achieved via non-parallel asymmetric apertures mimicking Babinet c-SRRs^37^. Extremely high polarization conversion efficiency has been achieved in the visible region via bilayers of complementary antenna-aperture arrays^38^. Dual-band asymmetric transmission has been achieved in the infrared region both for linear and circular polarization due to the coupling between bilayers of twisted complementary SRRs and gammadions forming a chiral metamaterial^39,40^. The additional degrees of freedom achievable in multilayer metasurfaces are particularly important for applications^41^. The empowered phenomena in multilayers involve near-field coupling, waveguiding and interference, which allow for spectral engineering, polarization manipulation, chirality effects and phase control^42^. As a result, multilayers are ideal candidates for fundamental studies on PT symmetric metamaterials^24–26^ as well as for designing elements of next-generation nanophotonic devices^43^.

In our previous studies, illumination by linearly polarized light was realized to compare the transmittance and reflectance of complementary convex and concave complex pattern configurations that can be fabricated by integrated colloid-sphere and interference lithography^44–46^. We demonstrated that in the case of linearly polarized light illumination, the convex transmittance subtracted from unity (reflectance) and the concave inverted rectified reflectance (rectified transmittance) extracted from the polarization unspecific power-outflow read-out, are complementary^44–46^. In our present study, the polarization specifically read-out transmittance signals of Babinet complementary single and multilayers illuminated by linearly and circularly polarized light are determined via Jones matrix elements to demonstrate metamaterial capabilities^47–49^. The multilayers can be fabricated using multistep e-beam lithography or the integrated lithography method described in our previous studies^50,51^. These 3D patterns are tuneable because of the high degrees of freedom of integrated lithography and compose metamaterials exhibiting extrinsic chirality and NIM, as demonstrated in this study.

## Methods

**Figure 1:**
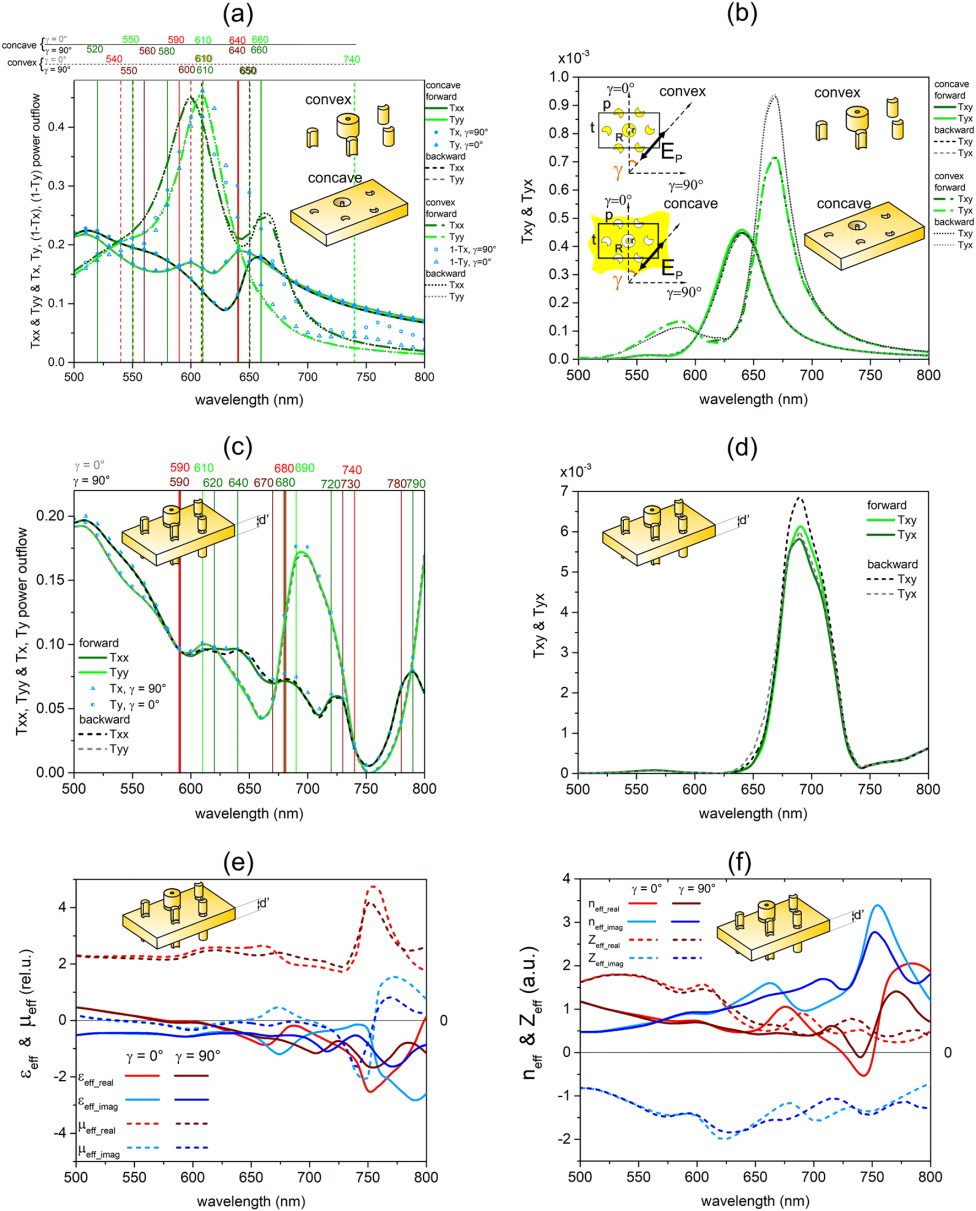
Optical responses and effective parameters: linearly polarized light illumination. (**a**, **c**) Comparison of copolarized (T_xx_ and T_yy_) and (**b**, **d**) cross-polarized (T_xy_ and T_yx_) transmitted signals, as well as total signals from polarization unspecific power-outflow read-outs (depicted with blue symbols): (**a**, **b**) convex and concave single layer (empty and filled), (**c**, **d**) multilayer (empty), for 90° (spheres) and 0° (triangles) azimuthal orientations. (**a**, **b**) Transmittance of single layer convex (subtracted from unity, dashed-dot and dot) and concave (continuous and dashed) 300-nm periodic patterns of miniarrays, the linearly polarized light propagates forward (green continuous and dashed-dot) and backward (grey dashed and dot) and the extrema in the convex (dashed) (concave-rectified (continuous)) absorptance (red) and total transmittance (green). (**c**, **d**) Transmittance of the multilayer, the linearly polarized light propagates forward (green continuous) and backward (grey dashed) and the extrema in the absorptance (red) and total transmittance (green). (**e** and **f**) Dielectric properties and index of refraction of the multilayer determined using a retrieval method based on S parameters extracted from illumination by linearly polarized light, in both azimuthal orientations.

**Figure 2:**
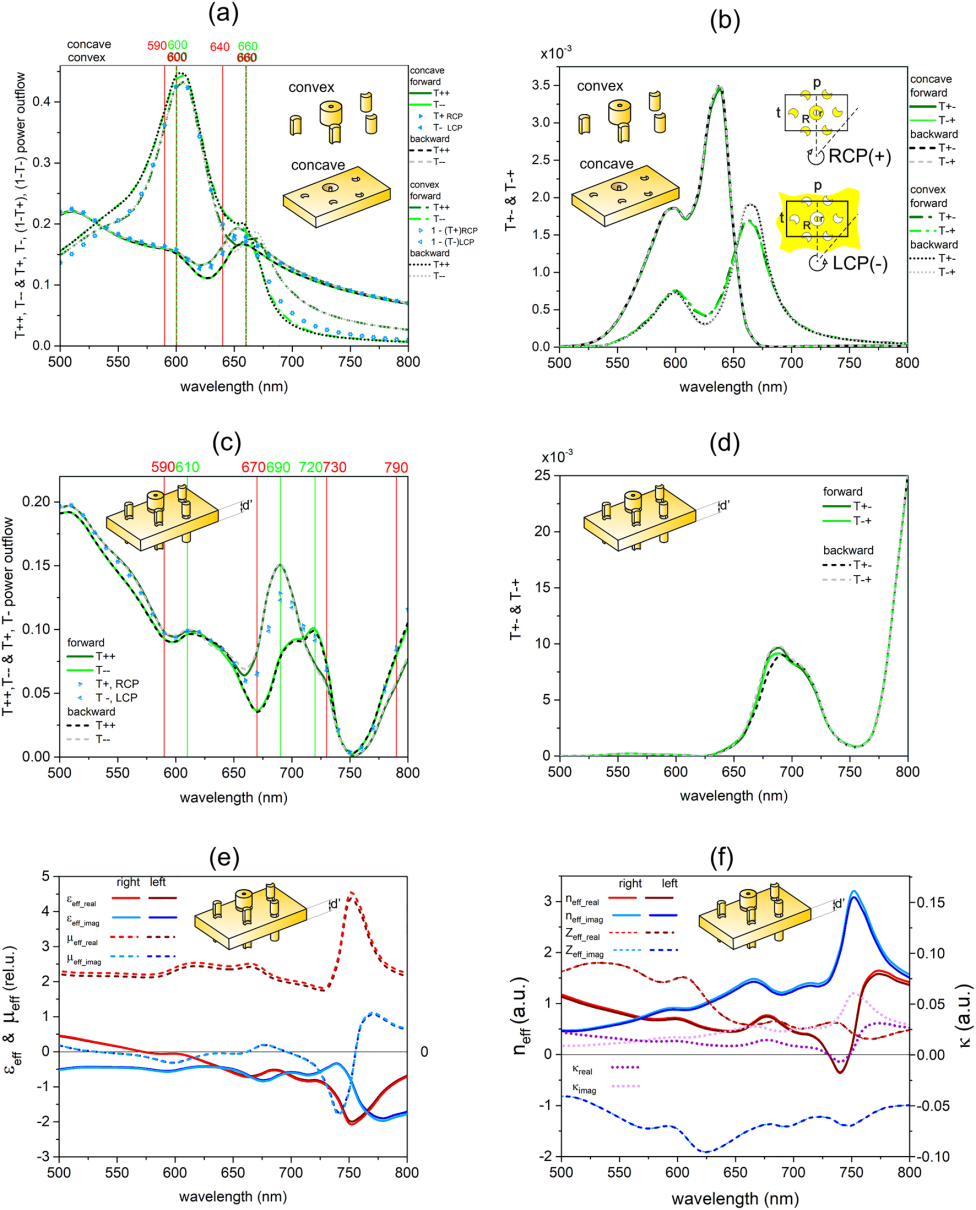
Optical responses and effective parameters: circularly polarized light illumination. (**a**, **c**) Comparison of copolarized (T_++_ and T_−−_) and (**b**, **d**) cross-polarized (T_+−_ and T_−+_) transmitted signals, as well as total signals from polarization unspecific power-outflow read-outs (depicted with triangular symbols): (**a**, **b**) convex and concave single layer (empty and filled), and (**c**, **d**) multilayer (empty), right and left tip for right and left handednesses. (**a**, **b**) Transmittance of single layer convex (subtracted, dashed-dot and dot) and concave (continuous and dashed) 300-nm periodic patterns of miniarrays, the circularly polarized light propagates forward (green dashed-dot and continuous) and backward (grey dot and dashed) and the extrema in the convex (dashed) (concave-rectified (continuous)) absorptance (red) and transmittance (green). (**c**, **d**) Transmittance of the multilayer, the circularly polarized light propagates forward (green continuous) and backward (grey dashed) and the extrema in the absorptance (red) and total transmittance (green). (**e** and **f**) Dielectric properties and index of refraction of the multilayer determined using a retrieval method based on S parameters extracted from illumination by circularly polarized light, for both handednesses.

Babinet complementary single-layer concave and convex complex patterns were inspected first. The *p* = 300-nm periodic rectangular patterns of convex and concave miniarrays, which are composed of a central nanoring (*R* = 25 nm outer, *r* = 5 nm inner radius) and satellite nanocrescents, can be directly fabricated by integrated lithography, as described in our previous studies^44–46,50,51^. The thickness of the gold film (*d* = 45 nm) embedding the apertures filled with material, identical with that in the surrounding medium, was equal to the height of convex nano-objects. These complex patterns were re-illuminated by linearly and circularly polarized light, and polarization-selective transmitted signal read-out was realized using the finite element method in the RF module of COMSOL Multiphysics (Fig. 1 and Fig. 2). The complete complementary optical responses, namely the transmittance and reflectance that were read-out polarization unspecifically, as well as the absorptance determined based on Joule heating, are presented in Supplementary Information (see Supplementary Fig. S1 and Fig. S2).

First single layer of 300-nm periodic patterns of complementary convex and concave miniarrays embedded into NBK7 medium were investigated (insets in Figs. 1a, b and 2a, b). Then, the metal film thickness and distance between consecutive layers in a multilayer made of convex–concave–convex miniarray patterns were optimized (*d*’ = 39 nm and *s* = 5 nm) to construct a 3D structure and to achieve NIM in the visible region (insets in Figs. 1c, d and 2c, d, see Supplementary Information Fig. S3a). A multistep e-beam or integrated lithography procedure is necessary, including lift-off stages to transfer the concave miniarrays into complementary convex ones, and a synchronization method is indispensable, to ensure vertically aligned stacks of miniarrays in the convex–concave–convex layers^50,51^.

The inspection of the rectified and unrectified concave transmitted signals, along with the convex transmittance, was performed for linearly and circularly polarized light illuminations. Rectification was performed by subtracting the signal of a continuous gold film, thereby computing the rectified transmittance (dT) as well as absorptance (dA) and reflectance (dR, shown in Supplementary Information). For linearly polarized light illumination, the transmittance signal was read-out polarization selectively for the 0° and 90° azimuthal orientations (Fig. 1a–d). These orientations correspond to U/C and C/U resonances on the almost horizontal convex/concave nanocrescents (see Supplementary Fig. S1a–c)^44–46^. The inspection of circularly polarized transmitted signals computed using the linear base representation of the Jones transmission matrix elements was also realized (Fig. 2a–d, Supplementary Fig. S2)^47–49^. This polarization-selective read-out allowed us to consider the existence of copolarized and cross-polarized transmitted signal components, to compare their azimuthal orientation dependence for linear polarization and handedness dependence (chirality) for circular polarization, as well as to uncover potential asymmetric transmission (propagation direction dependence for the same original polarization) phenomena that might be important in various applications (Figs. 1a–d, 2a–d)^33,38,47–49^.

Convex and concave miniarrays in single-layer periodic patterns were illuminated by linearly and circularly polarized light that propagated forward (+ z direction) and backward (-z direction). The spectral features appearing in copolarized and cross-polarized transmitted signals were compared with the extrema in the convex absorptance and total transmittance, as well as with the extrema in the concave rectified absorptance and rectified total transmittance, respectively (Figs. 1a, b, 2a, b).

Then, a multilayer embedded into the NBK7 medium was illuminated by linearly and circularly polarized light that propagated forward and backward. The spectral features appearing in copolarized and cross-polarized transmitted signals were compared with the extrema in the absorptance and total transmittance (Figs. 1c, d, 2c, d).

The effective constitutive parameters were extracted using the retrieval method developed for reciprocal slabs, and the potential to achieve the NIM phenomenon was also inspected (Figs. 1e, f, 2e, f)^5–10^. For circularly polarized light illumination, handedness dependence that can manifest itself in optical activity and circular dichroism was qualified by the chirality factor^18–26^. Accordingly, besides the dielectric permittivity, permeability, and refractive index, the chirality factor was also determined for circularly polarized light illumination (Fig. 2e, f).

**Figure 3:**
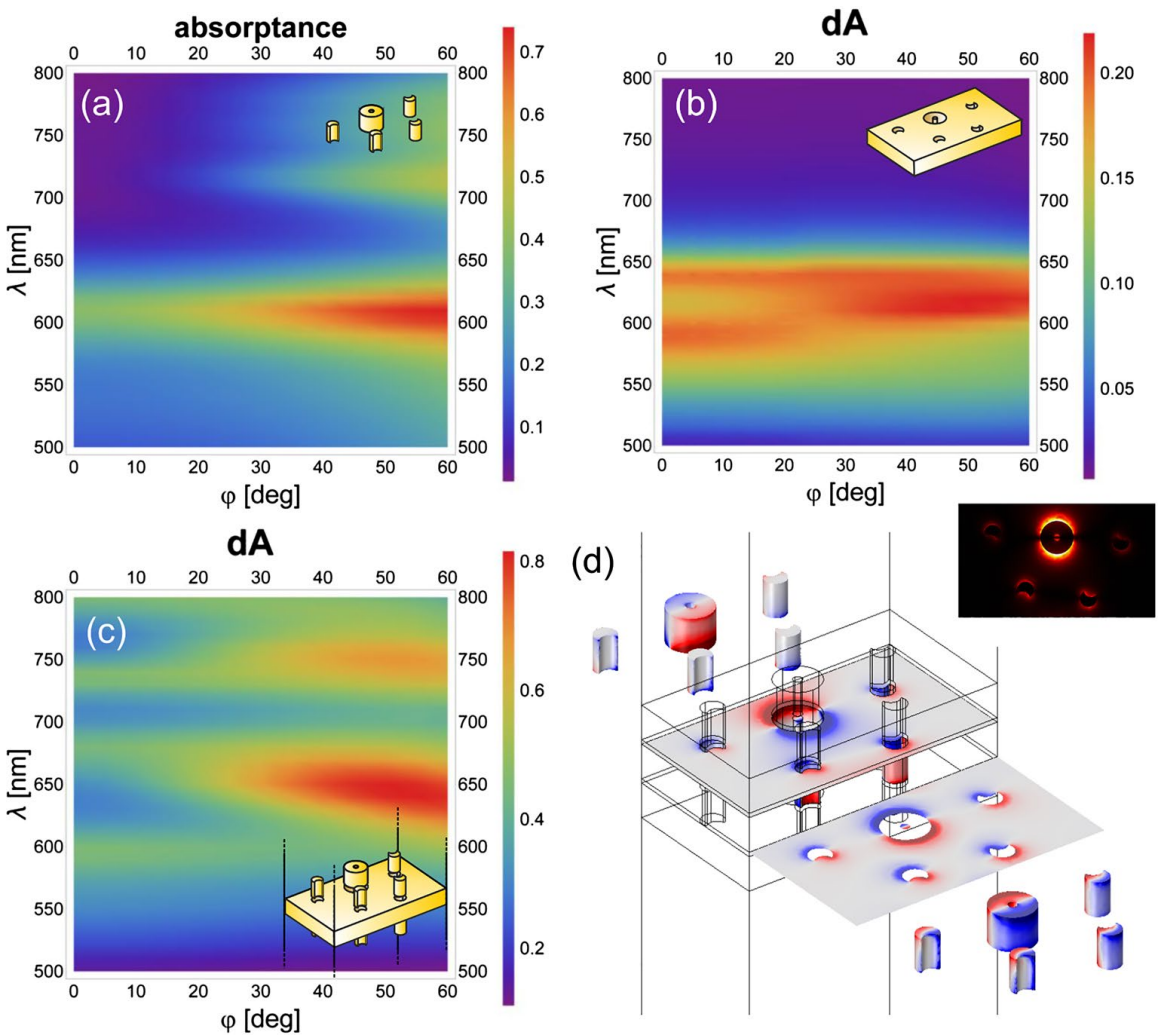
Dispersion map and excited modes: linearly polarized light illumination in 0° azimuthal orientation. Single layer of (**a**) convex and (**b**) concave miniarrays, and (**c**) multilayer of convex–concave–convex miniarrays. (**d**) Charge distribution at negative index on the multilayer (see Supplementary Video S1 online); inset: abs E_z_ field component.

**Figure 4:**
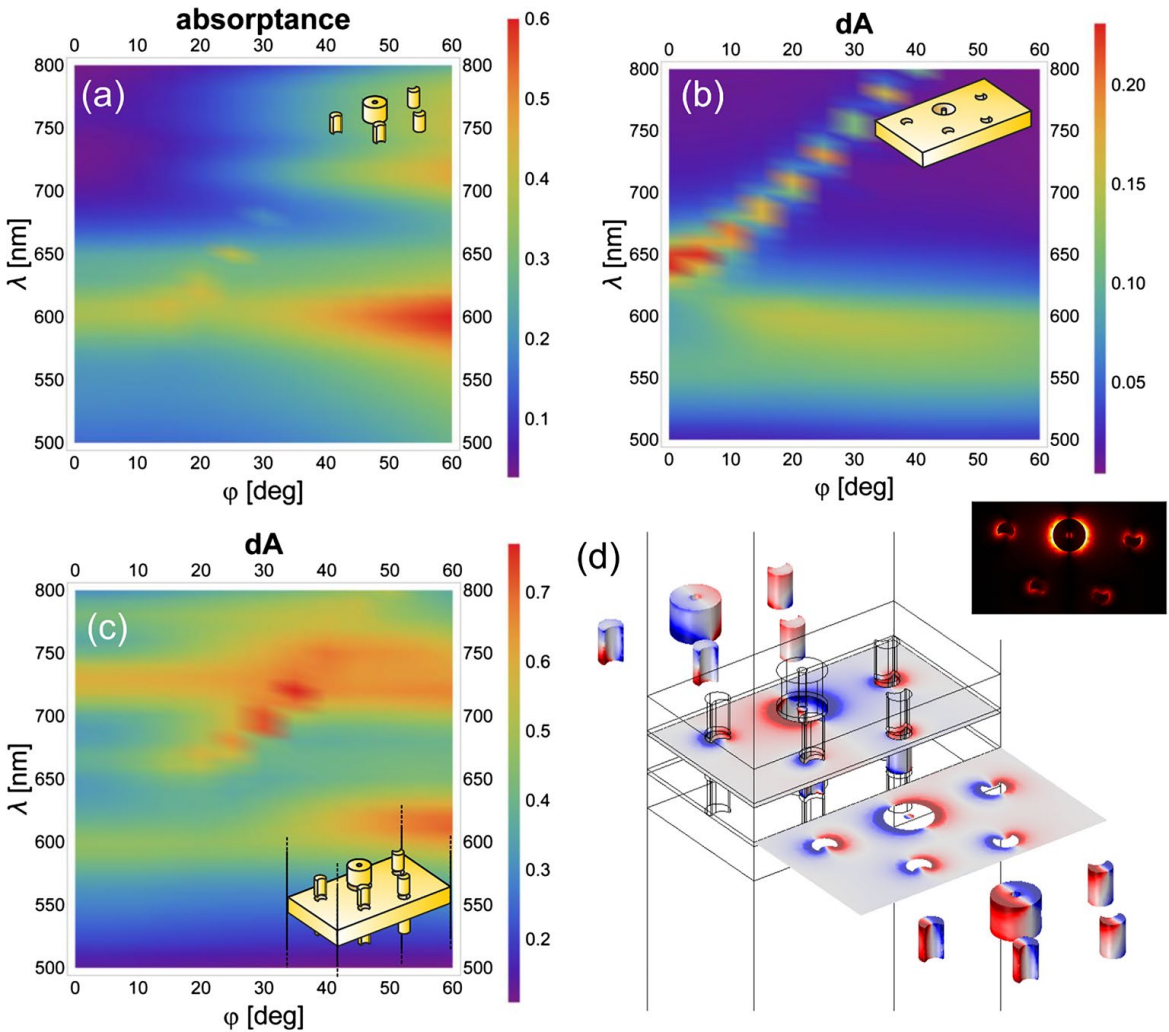
Dispersion map and excited modes: linearly polarized light illumination in 90° azimuthal orientation. Single layer of (**a**) convex and (**b**) concave miniarrays, and (**c**) multilayer of convex-concave-convex miniarrays. (**d**) Charge distribution at negative index on the multilayer (see Supplementary Video S2 online); inset: abs E_z_ field component.

**Figure 5:**
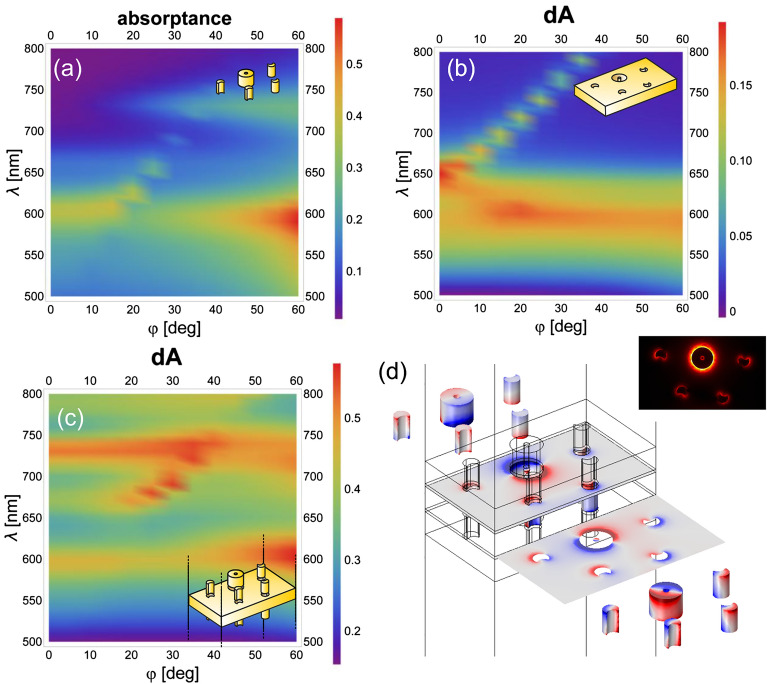
Dispersion map and excited modes: illumination with right-handed (RCP: +) circularly polarized light. Single layer of (**a**) convex and (**b**) concave miniarrays, and (**c**) multilayer of convex–concave–convex miniarrays. (**d**) Charge distribution at negative index on the multilayer (see Supplementary Video S3 online); inset: abs E_z_ field component.

**Figure 6:**
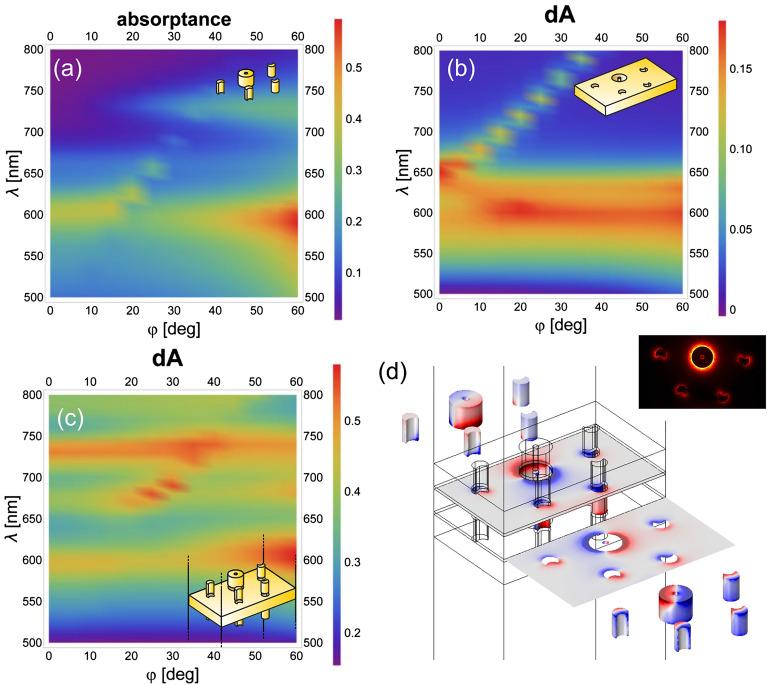
Dispersion map and excited modes: illumination with left-handed (LCP: −) circularly polarized light. Single layer of (**a**) convex and (**b**) concave miniarrays, and (**c**) multilayer of convex–concave–convex miniarrays. (**d**) Charge distribution at negative index on the multilayer (see Supplementary Video S4 online); inset: abs E_z_ field component.

The dispersion characteristics in absorptance were mapped for linearly (Figs. 3a–c, 4a–c) and circularly (Figs. 5a–c, 6a–c) polarized light illuminations, because it exhibits enhancement in tilting independent and dependent bands, when localized surface plasmon resonances (LSPR) and surface plasmon polariton (SPP) modes are excited, respectively^52^. The bands are depicted in the absorptance for the convex single layer and in the rectified absorptance for the concave single layer and the multilayer. Also for the multilayer the absorptance of a continuous metal film, but of the optimized *d*’ thickness, was subtracted. The near-field and charge distribution was inspected at the spectral locations corresponding to the NIM phenomenon (Figs. 3d and 4d, 5d and 6d show the illumination with linearly polarized light in the 0° and 90° azimuthal orientation, with right-handed and left-handed circularly polarized light, respectively, Supplementary Videos 1–4) as well as at the spectral location of the most pronounced handedness dependence (see Supplementary Information Fig. S3, Supplementary Videos 5, 6), to uncover the nanophotonic origin of these phenomena. Finally, the accompanying magnetic dipoles’ dynamics was studied (see Supplementary Information Fig. S4).

## Results

### Single layer illumination by linearly polarized light

All types of plasmonic resonances manifest themselves in absorptance peaks, and the transmittance and reflectance extrema are slightly shifted compared to the absorptance peaks, in accordance with the literature^52^. The results detailed in our recent papers prove that there is good (reasonable) complementarity between the convex subtracted-transmittance (reflectance) and the concave rectified-inverted reflectance (rectified transmittance), respectively (see Supplementary Information, Fig. S1a–c)^44–46^. In the 90°/0° azimuthal orientation of the convex/concave single layer, the ring coincident with the mixed C1–C2 resonances and the C1 resonance are at play, whereas in the 0°/90° azimuthal orientation, the U resonance can be excited, which is coupled with SPP modes on the concave layer. In the case of linearly polarized light illumination, both the convex and concave 300-nm periodic patterns result in copolarized and cross-polarized light components, as the T_xy_ and T_yx_ signals are nonzero (Fig. 1a, b). The copolarized components and total transmitted signals differ only slightly, because of the tiny amplitude of the cross-polarized components. The sum of the copolarized and cross-polarized components equals to the total transmitted signal, namely, the polarization unspecifically read-out power-outflow.

The copolarized transmittance is single/double peaked for the convex pattern, whereas it exhibits two smaller /one larger modulations for the concave pattern in the 0°/90° azimuthal orientation. In the copolarized transmittance, both the convex and concave patterns exhibit azimuthal orientation dependence, because T_xx_ significantly differs from T_yy_. In the case of the convex pattern the distinct 0.143 and 0.154 maximal differences are observable at 670 nm, where a local maximum appears in the subtracted T_xx_ transmission signals in the case of illumination by forward and backward propagating light. In the case of the concave pattern the (analogous 0.0787 and 0.0793) maximal differences appear at 630 nm in the case of forward and backward propagating light, where the global minimum in T_xx_ transmission monitored in the 90° azimuthal orientation coincides with the inflection preceding the global maximum in T_yy_ transmission monitored in the 0° azimuthal orientation.

The cross-polarized signal transmitted on the convex pattern shows a local—global maximum pair, whereas it exhibits a small shoulder and a single maximum on the concave pattern. The cross-polarized transmittance does not show azimuthal orientation dependence for either of the convex or concave patterns, because both the local—global maximum and the shoulder—global maximum pairs coincide for the two orthogonal original polarization orientations. Namely, in the convex cross-polarized signal, the local—global maximum pair appears at the same 590 nm and 670 nm locations in the T_xy_ and T_yx_ signals in both the forward and backward illumination light propagation directions. In comparison, the cross-polarized signal of the concave pattern exhibits a shoulder—global maximum pair at 560 nm and 640 nm locations that also do not depend on the azimuthal orientation for either propagation direction.

There is no propagation direction dependence in the copolarized transmitted signal for either azimuthal orientations of the concave pattern. The T_xx_ monitored in the 90° azimuthal orientation considerably differs for the two propagation directions through the convex pattern, and the maximal difference (0.0134) is taken on at 670 nm, while a three-times smaller (0.0053) maximal difference is taken on at 620 nm in T_yy_ in the 0° azimuthal orientation. Moreover, there is no noticeable difference between the cross-polarized signals monitored for the illumination of the concave pattern with forward and backward propagating linearly polarized light, whereas the cross-polarized signal exhibits almost an order-of-magnitude larger but still weak asymmetric transmission in the case of the convex pattern.

The global maximum of 2.11 × 10^–4^ in the difference between the x-to-y conversion in forward and backward propagating transmitted signal components (|T_yx_forward_−T_yx_backward_|) appears at 670 nm. Analogously, the maximal difference of 2.15 × 10^–4^ in the y-to-x conversion between backward and forward propagating components (|T_xy_backward_−T_xy_forward_|) is taken on at 670 nm. The location of the maximal asymmetric transmission through the convex pattern is coincident in T_xx_ and in the cross-polarized signals.

### Multilayer illumination by linearly polarized light

On the multilayer illuminated by linearly polarized light in the 0° azimuthal orientation U (C) resonant modes are excited, whereas in the 90° azimuthal orientation C (U) resonant modes are excited on the convex (concave) layers. The spectral bands of the ring resonance coincident with the mixed C1–C2 resonances and of the C1 resonance overlap with the bands of the U resonance and of the coupled SPP modes, in couples. The overlap of these modes promotes coupling between the layers, and the resulting bands appear at forward-shifted spectral locations that slightly differ in absorptance and transmittance signals. In addition to these extrema, absorptance peaks appear at 740 nm in the 0° azimuthal orientation as well as at 730 nm and 780 nm in the 90° azimuthal orientation (see Supplementary Information Fig. S1d).

Furthermore, in case of linearly polarized light illumination, the multilayer results in copolarized and cross-polarized transmitted signal components, as T_xy_ and T_yx_ signals are nonzero (Fig. 1c, d). The copolarized components and complete transmitted signals differ slightly, based on the small amplitude of the cross-polarized components. The sum of the copolarized and cross-polarized transmitted signal components equals to the polarization unspecifically read-out total power-outflow for both azimuthal orientations.

The copolarized transmittance is multiple-peaked in both azimuthal orientations of the multilayer (Fig. 1c). The multilayer also exhibits azimuthal orientation dependence in the copolarized component, since T_xx_ significantly differs from T_yy_. In case of the inspected multilayer the analogous 0.1129 and 0.1124 maximal difference is observable at 700 nm in case of forward and backward propagation direction. This occurs close to the location, where a local minimum develops in T_xx_ signals monitored in the 90° azimuthal orientation and a large local maximum appears in T_yy_ signal monitored in the 0° azimuthal orientation. The location of the maximal difference is forward-shifted in the multilayer compared to those in single convex (670 nm) and concave (630 nm) composing layers. The single-layer specific transmittance peaks are accompanied by a shoulder and a local maximum (730 nm) in the corresponding copolarized transmittance signals in the 0° and 90° azimuthal orientations, respectively. Similarly, a shoulder and a local maximum (720 nm) are observable in the case of polarization unspecific read-out in the 0° and 90° azimuthal orientations, respectively (see Supplementary Information, Fig. S1d).

The cross-polarized transmittance exhibits a small local maximum and a global maximum (Fig. 1d). The cross-polarized transmittance does not show azimuthal orientation dependence for the multilayer, since the local–global maximum appears at the same 560–690 nm location both in the T_xy_ and T_yx_ signals, both for forward and backward light propagation directions. The local maximum in the cross-polarized signal is backward-shifted compared to the local maximum (590 nm) (coincident with the shoulder (560 nm)) in the cross-polarized signal of the convex (concave) layer, whereas the global maximum is forward-shifted compared to the global maxima in cross-polarized signals of the convex (670 nm) and concave (640 nm) composing layers.

There is no significant propagation direction dependence in either of the copolarized or cross-polarized transmitted signals, namely, the transmission almost equals for the illumination by forward and backward propagating linearly polarized light. The maximal difference between the forward and backward propagating T_xx_ and T_yy_ copolarized signals is almost three-times smaller (0.0048 at 660 nm) and the same (0.005 at 680 nm), while the difference between the T_xy_ and T_yx_ cross-polarized signals is three-times and two-times larger, when compared to the difference between analogous signals transmitted through the single convex layer. The considerable asymmetric transmission observable for the T_xx_ copolarized signal in the case of single-layer convex pattern is not preserved, despite that two convex layers surrounds one single-layer concave pattern.

### Single layer illumination by circularly polarized light

On the single-layer convex pattern, there is no difference between the spectral locations of the absorptance and subtracted transmittance peaks in the case of circularly polarized light illumination with different handednesses. On the single layer concave pattern there is a small difference between the peaks in the rectified absorptance and transmittance for both handednesses (see Supplementary Information, Fig. S2a–c). The convex subtracted transmittance (reflectance) and concave inverted-rectified reflectance (rectified transmittance) extracted from the polarization unspecific power-outflow read-out in the case of circularly polarized light illumination are not perfectly complementary. The type of extrema is interchanged for convex and concave patterns, and there is a spectral shift as well (see Supplementary Information, Fig. S2a–c). There is a small difference between the total transmitted signals for the two handednesses, when polarization unspecific read-out is applied (Fig. 2a).

In the case of circularly polarized light illumination, both the convex and concave 300-nm periodic patterns result in copolarized and cross-polarized light components, since T_+-_ and T_-+_ signals are nonzero (Fig. 2b). The copolarized components and the complete transmitted signals differ only slightly, because of the small amplitude of the cross-polarized components (Fig. 2a). A larger difference is predicted between the transmittances of left and right-handed circularly polarized light, when the copolarized and cross-polarized signal components are computed using the linear base representation of the Jones transmission matrix elements (Fig. 2a).

The copolarized transmittance exhibits extrema in two spectral intervals for both handednesses, the signal modulation is more pronounced in the case of right/left-handed circular polarization for forward/backward propagating light illumination of the convex pattern, whereas the degree of modulation is very similar for all inspected configurations of the concave pattern. The copolarized transmitted spectra both for the convex and concave patterns show handedness dependence, as T_++_ significantly differs from T_−−_. In the case of the convex pattern, the distinct 0.056 and 0.064 maximal difference is observable for the forward and backward propagation directions at 680 nm, which is after the transmittance modulations related to nano-object resonances. In the case of the concave pattern the handedness dependence of the transmittance is significant between 600 and 700 nm, the analogous 0.0322 and 0.0318 maximal difference appears at 640 nm in the case of forward and backward propagating light, but the difference disappears at 740 nm.

The cross-polarized transmittance of the convex pattern shows a local–global maximum pair that is coincident for both handednesses, even though its amplitude depends slightly on the propagation direction. In comparison, the cross-polarized transmittance of the concave pattern exhibits a local–global maximum pair that is coincident for both handednesses, and is identical for both propagation directions. The complete cross-polarized transmitted spectrum does not show handedness dependence for either of the convex or concave pattern. In the convex pattern a local–global maximum pair appears at the same 600 nm and 670 nm locations in the T_+−_forward_ and T_−+_forward_ transmittance for both handednesses in the case of illumination by forward propagating circularly polarized light. A similar local–global maximum pair is observable at 600 nm and at a slightly backward shifted 660-nm location in the T_+−_backward_ and T_−+_backward_ transmittances for both handednesses in the case of illumination by backward propagating circularly polarized light. In comparison, for the concave pattern the cross-polarized transmitted signal exhibits a local–global maximum pair at the same 600–640 nm locations, which does not depend on the handedness in the case of illuminations either with forward or backward propagating light.

In the copolarized transmitted signals that are expected to be analogous by means of reciprocity in this structure configuration, there is no propagation direction dependence for the concave pattern, namely T_++_forward_ = T_−−_backward_, and vice versa. For the convex pattern T_−−_forward_ ≈ T_++_backward_, while T_++_forward_ and T_−−_backward_ differ more noticeably, the maximal difference (0.00841 and 0.01028) appears at 670 nm. The |T_++/−−_forward_—T_++/−−_backward_| difference (considerable as an asymmetric dichroism) between the forward and backward propagating copolarized transmitted signals is slightly larger/smaller both on the concave and convex pattern, than the dichroism for forward/backward propagation.

Moreover, there is no noticeable difference between the analogous forward and backward propagating cross-polarized components for the concave pattern, whereas the cross-polarized component exhibits a weak asymmetric transmission in the case of the convex pattern also for circularly polarized light illumination. The small 2.66 × 10^–4^ maximal difference between the left-to-right forward and right-to-left backward components (|T_+−_forward_–T_−+_backward_|) as well as between the left-to-right backward and right-to-left forward (|T_+−_backward_–T_−+_forward_|) components appears at 670 nm. Similar small difference is reached at 670 nm between the forward and backward propagating cross-polarized signals for the same original polarization, (|T_−+_forward_–T_−+_backward_|) and (|T_+−_forward_–T_+−_backward_|). The location of the asymmetric transmission is coincident in T_++_ and T_−−_ as well as in the cross-polarized signals of the convex pattern.

### Multilayer illumination by circularly polarized light

The responses of the multilayer to circularly polarized light illumination are very similar to the sum of responses from linearly polarized light illumination. On the multilayer, the spectral maximum pair appearing at the ring resonance coincident with the mixed C2–C1 resonances and the C1 resonance, as well as at the U resonance and the coupled SPP mode extrema pairs, are overlapping, in couples. The overlapping of these modes facilitates efficient coupling between consecutive layers, and the resulting bands (600 nm and 660 nm) are slightly forward-shifted compared to the extrema on the single layer. The spectral locations again slightly differ in absorptance and transmittance signals. In addition to these single-layer-related extrema, absorptance peaks appear at 730 nm and 790 nm for both handednesses (see Supplementary Information Fig. S2d). The former peak is accompanied by a shoulder at 730 nm in the corresponding polarization unspecifically read-out transmittance signals (Fig. 2c).

The illumination of the multilayer by circularly polarized light results in copolarized and cross-polarized light components as well, as T_+−_ and T_−+_ signals are nonzero (Fig. 2c, d). The copolarized components and complete transmitted signals computed using the linear base representation of the Jones transmission matrix elements significantly differ, because of the considerably larger cross-polarized components (Fig. 2c).

The copolarized transmittance exhibits extrema in multiple spectral intervals for both handednesses, and the signal modulation is different in the case of right and left-handed polarization, similar to the composing convex and concave patterns, but equals for the two propagation directions. A significantly larger difference is predicted between the transmittances of left and right-handed circularly polarized light, when the transmitted copolarized and cross-polarized signal components are computed using the linear base representation of the Jones transmission matrix elements. Based on the Jones matrix element calculations, the multilayer exhibits pronounced handedness dependence in the copolarized transmitted signal, as T_++_ significantly differs from T_−−_. For the inspected multilayer the analogous 0.0814 and 0.0834 maximal difference is observable at 680 nm in case of forward and backward propagating light. The location of the maximal handedness sensitivity is after the transmittance modulations related to single layers, coincides with the location of maximal handedness dependence for the convex layer, but precedes the global minimum arising at 750 nm in the transmittance signal of the multilayer for both handednesses. For the multilayer the handedness dependence is significant throughout in the interval of 620–710 nm, rapidly decreases and approaches zero, but it is restored between 730 and 750 nm with the same sign. At the novel maximum appearing in the absorptance of the multilayer, an inflection is observable in the right/left handed copolarized forward/backward transmitted signal, whereas a local maximum appears in the left/right handed copolarized forward/backward transmitted signal (Fig. 2c). In contrast, a shoulder is observable in the case of polarization unspecific read-out for both handednesses (see Supplementary Information Fig. S2d).

The cross-polarized transmittance shows a local maximum and a global maximum coincident for both propagation directions and handednesses and it is significantly larger than the cross-polarized transmittance maximum on single layers (Fig. 2d). The cross-polarized transmittance does not exhibit handedness dependence for either of the propagation directions. The local and global maximum pair appears at the same 560–680 nm location for both handednesses. The local extremum is backward-shifted, whereas the global maximum is forward-shifted compared to the similar extrema in cross-polarized signals in the case of convex (600–670/660 nm) and concave single layers (600–640 nm).

There is no propagation direction dependence for either of the copolarized or the cross-polarized transmitted signal components that are expected to be analogous by means of reciprocity in this structure configuration, namely T_++_forward_ ≈ T_−−_backward_, and T_−−_forward_ ≈ T_++_backward_, the maximal difference (0.0047 and 0.0038) appears at 660 nm and 640 nm, while T_+−_forward_ = T_−+_backward_ as well as T_−+_forward_ = T_+−_backward_). The maximal difference between the analogous forward and backward propagating copolarized signals (|T_++/−−_forward_–T_−−/++_backward_|) is two-/three-times smaller, while the maximal difference between the cross-polarized signals (|T_+−/−+_forward_–T_−+/+−_backward_|) is two-times and four-times larger, when compared to the difference between analogous signals transmitted through the single convex layer.

The maximal difference between the forward and backward propagating copolarized transmitted signals (|T_++/−−_forward_–T_++/−−_backward_|, considerable as an asymmetric dichroism) is slightly larger/smaller at 680 nm, than the dichroism for forward/backward propagation. The |T_++/−−_forward_–T_++/−−_backward_| difference between the forward and backward propagating copolarized signals is commensurate with the sum of differences in composing layers, while the small (|T_−+_forward_–T_−+_backward_|)/(|T_+−_forward_–T_+−_backward_|) difference between the cross-polarized signals is several-times larger, when compared to the asymmetric transmission signals on the single convex layer.

### Effective optical parameters, chirality and NIM phenomenon: linearly polarized light illumination

In the case of illumination with linearly polarized light, the imaginary part of the effective permittivity (ε_eff_imag_) is negative in the complete inspected interval, and also the real part (ε_eff_real_) becomes negative at wavelengths greater than 575 nm in both azimuthal orientations. The local minima appear in the real part of the effective permittivity at 660 nm and 750 nm/705 nm and 750 nm, while in the imaginary part of the effective permittivity at 675 nm and 790 nm/715 nm and 770 nm in the 0°/90° azimuthal orientation. Moreover, a shoulder is observable at 760 nm in the 0° azimuthal orientation both in the real and imaginary parts. In contrast, only the imaginary part of the effective permeability (μ_eff_imag_) takes on negative values in the inspected interval, and there are two local minima at 600 nm and 750 nm/740 nm in the 0°/90° azimuthal orientation (Fig. 1e). The overlapping regions of the negative ε_eff_real_, ε_eff_imag_ and μ_eff_imag_ are coincident with the narrow spectral region (centred at 740 nm), where the real part of the effective index of refraction (n_eff_real_) takes on a negative value of − 0.6 and − 0.1 in the 0° and 90° azimuthal orientations of the multilayer, respectively (Fig. 1f).

### Effective optical parameters, chirality and NIM phenomenon: circularly polarized light illumination

In the case of circularly polarized light illumination the imaginary part of the effective permittivity (ε_eff_imag_) is negative in the complete inspected interval, and also the real part of the effective permittivity (ε_eff_real_) becomes negative throughout a wide spectral region above 575 nm. The local minimum appears at 670 nm and 675 nm in the real and imaginary parameters, followed by a global minimum at 750 nm and 780 nm, respectively. However, only the imaginary part of the effective permeability (μ_eff_imag_) takes on negative values in the inspected interval, there are local and global minima at 600 nm and 740 nm (Fig. 2e).

The overlapping regions of the negative ε_eff_real_, ε_eff_imag_ and μ_eff_imag_ are coincident with the narrow spectral region (centred at 740 nm), where the real part of the effective index of refraction (n_eff_real_) takes on a negative value of − 0.36. This proves that a negative index is achieved both for right and left-handed circularly polarized light illumination. At this spectral location the real part of the chirality factor (κ_real_) is also negative. The small nonzero (− 0.007) value of the chirality factor indicates that the multilayer possesses external chirality, but the NIM phenomenon does not have a chirality origin in the classical sense (Fig. 2f).

The inspection of the optical responses at the spectral location of the NIM phenomenon shows that the difference between the transmissions of circularly polarized light of different handednesses approaches zero; however, it remains small, the forward and backward transmitted signals almost coincide independent of handedness, the copolarized transmitted signal components approach the cross-polarized ones, moreover all transmission signals approach zero. The close-to-switching in the sign of the difference between left and right handed copolarized transmission indicates that the spectral location of the NIM phenomenon can be predicted by computing the copolarized (and cross-polarized) transmitted signal components for circularly polarized light illumination using the linear base representation of the Jones matrix elements. The dispersion characteristics and the charge distribution were inspected to uncover the nanophotonic origin of the negative index of refraction.

### Dispersion characteristics of single layers and of the multilayer illuminated by linearly and circularly polarized light

When the multilayer is illuminated by linearly polarized light in the 0° azimuthal orientation, U resonances on convex nano-objects interact with C resonances on concave nano-objects. In comparison, in the 90° azimuthal orientation C resonances on convex nano-objects interact with U resonances on concave nano-objects coupled with the propagating SPP modes on the patterned metal film^44–46^.

The dispersion characteristics of all inspected layer configurations show a localized plasmon resonance-related tilting-independent flat bands (Figs. 3, 4, 5, 6a, b). Accordingly, LSPR-related peaks appear at perpendicular incidence, e.g., at 610 nm/600 nm and –/650 nm in the absorptance of the single-layer convex pattern and at 590 nm/560 nm and 640 nm/– in the rectified absorptance of the single-layer concave pattern illuminated by linearly polarized light in the 0°/90° azimuthal orientation (Figs. 3, 4a, b). On the dispersion map of the convex single layer in the 0° azimuthal orientation, there is no scattered mode-related band that could interact with this LSPR band at perpendicular incidence (Fig. 3a). Although, in the 90° azimuthal orientation, such a branch appears close to the LSPR band (~ 600 nm) but intersects it at ~ 30° tilting (Fig. 4a). In addition to this, a weak flat band is recognizable at ~ 740 nm/730 nm at similar tilting in the 0°/90° azimuthal orientations, but this also cannot be excited at perpendicular incidence efficiently (Figs. 3a, 4a). On the dispersion map of the concave single layer, there are only LSPR-related flat bands in the 0° azimuthal orientation at 590–640 nm. In contrast, in the 90° azimuthal orientation the well-defined propagating plasmonic mode-related band causes an anti-crossing manifesting itself in the coupled LSPR–SPP branches. These branches are related to the modes at 560–640 nm at perpendicular incidence, as described in Supplementary Information (Figs. 3b, 4b, see Supplementary Information Figs. S1a-c).

The circularly polarized light illumination of the convex single layer results in similar bands (600–660 nm) handedness independently and in relative strengthening of the flat band at ~ 740 nm/730 nm at transitional tilting in case of right/left-handed illumination, and facilitates its excitation already at smaller ~ 20° incidence angle (Figs. 3a and 4a to Figs. 5a and 6a). The circularly polarized light illumination of the concave single layer results in a relative strengthening and widening of the LSPR and the coupled bands (former 590/560 nm–640 nm) at their anticrossing (< 10° tilting), independent of handedness (Figs. 3b and 4b to Figs. 5b and 6b).

When these layers are integrated into a multilayer in convex–concave–convex succession, the originally dominant bands forward shift to ~ 600–680 nm and 600–670 nm in the rectified absorptance in the case of linearly polarized light illumination in the 0° and 90° azimuthal orientation, respectively (Figs. 3c and 4c). Similar bands are shifted to ~ 600–670 nm in the rectified absorptance in the case of circularly polarized light illumination for either handedness (Figs. 5c and 6c).

The additional LSPR-related flat band that is weak in single layers, is significantly/moderately strengthened for illumination of multilayers with linearly polarized light in the 0°/90° azimuthal orientation, and is considerably strengthened for illumination of multilayers with circularly polarized light of both handednesses.

As a result, additional resonance peak appears at perpendicular incidence at 730 nm (except in the 0° azimuthal orientation, where it appears at 740 nm) (Figs. 3, 4, 5, 6c). The corresponding band seems to interact predominantly with the SPP band at larger tilting, rather than the LSPR at 560 nm in the 90° azimuthal orientation. Moreover, the coupled LSPR and SPP modes supported by the concave single layer for linearly polarized light illumination in the 90° azimuthal orientation and circularly polarized light illumination seem to be de-coupled. This interaction governs the spectrum at perpendicular incidence despite the strongest coupling between the novel LSPR mode at 730 nm and the propagating SPP mode occurring at a tilting greater than 30° (Figs. 4, 5, 6c).

Considering that the single-layer convex pattern shows a flat band at approximately 740 nm/730 nm at larger tilting, in the novel coupled modes appearance a mode localized on the convex nano-objects is crucial.

There is only a small difference between the dispersion characteristics mapped by linearly polarized light in the 90° azimuthal orientation and by circularly polarized light of either handednesses (Fig. 4a–c to Figs.5a–c, 6a–c). The stronger/weaker negative index appears at the long-wavelength side (740 nm) of the novel LSPR/coupled modes for linearly polarized light illumination in the 0°/90° azimuthal orientation, and the intermediate negative index is in accordance with their averaged impact for circularly polarized light illumination of both handednesses. To determine the origin of the flat band, the charge distribution was inspected on the 3D nano-objects in three consecutive layers.

### Coupled modes on the multilayer in case of linearly and circularly polarized light illumination

At the negative index phenomenon-related extrema that arise, when illuminated by linearly polarized light, a predominantly dipolar charge distribution develops, and quadrupoles appear only intermittently on the central nanoring in the lower convex and middle concave layers, both in the 0° and 90° azimuthal orientations (Figs. 3d, 4d, see Supplementary Videos S1 and S2). The main difference between the charge distributions arising in the two azimuthal orientations is that the dipolar charge separation occurs predominantly along the **E**-field oscillation direction, i.e., along the y and x axes in the case of 0° and 90° azimuthal orientation, respectively.

The inspection of the time-evolution shows that on the lower convex layer, the charge distribution is predominantly dipolar on the nanoring and the 3D dipoles on the nanocrescents are synchronous with it. Intermittently, a quadrupolar charge distribution evolves on the nanoring caused by the forward propagating reversal charge separation, as long as the rotation/turning of the dipole occurs on its upper surface in the 0°/90° azimuthal orientation. Until this moment the 3D charge separation on the nanocrescents remains reversal (synchronous) with the charge separation on the upper (lower) nanoring surface. The main difference in the 90° azimuthal orientation is that turning rather than rotation occurs on the convex nanoring and quadrupoles also appear on the convex nanocrescents before the moment of charge polarity turning on the nanoring.

The 3D distribution is predominantly dipolar/intermittently quadrupolar on the concave nanoring in the 0°/90° azimuthal orientation and is accompanied by reversal 3D dipolar separation on the concave nanocrescents. When a 3D tilted dipole/quadrupole appears on the nanoring due to the rotation (turning)/turning (rotation) of the dipole on its lower (upper) surface, the 3D dipolar charge separation on the nanocrescents becomes synchronous (remains reversal) with the charge separation on the lower (upper) nanoring surface. The main difference in the 90° azimuthal orientation is the appearance of a quadrupole on the nanoring for a longer time interval caused by the reversal phase of dipoles on the lower and upper surfaces and the co-existence of the periodic charge modulation due to the coupled SPP modes. On the lower (upper) surface of the middle concave layer, the charge separation is reversal compared to that on the upper (lower) surface of the neighbouring bottom/top convex layers.

On the upper convex layer in the 0° azimuthal orientation, the charge distribution remains dipolar on the nanoring, and the 3D dipoles on the nanocrescents are reversal compared to it, except for the short time interval corresponding to the turning.

In the 90° azimuthal orientation, the dipolar charge separation is tilted rather than horizontal on the nanocrescents. Because these 3D dipoles have the same charge as the predominant mode on the neighbouring side of nanoring, an extended dipole appears on the miniarray. The charge separation on the lower surface of the 3D convex nano-objects is reversal compared to the charge separation on the upper surface of the concave layer in both azimuthal orientations (see a segments in Supplementary Videos S1 and S2).

Considering that the negative index is more pronounced in the 0° azimuthal orientation, either the appearance of quadrupoles (tilted dipoles) on the nanocrescents in the lower (upper) convex layer or the coexistence of the propagating SPP-related periodic modulation on the middle concave layer is not advantageous.

However, the main advantage of the 0° azimuthal orientation is the rotation (turning) of the electric dipole on the lower (upper) surface of the concave nanoring, where the charge density is larger (smaller). As a result, a predominant rotating-tilted electric dipole and by implication a rotating-tilted closed loop of the **D** displacement vector appears. The rotating-tilted closed** D** loop is observable in a time-varying vertical plane (e.g. x–z and y–z planes: D_xz_, D_yz_), and **H**-field vortices are observable in the plane orthogonal to the **D** loop (e.g. y–z and x–z planes: corresponding H_yz_, H_xz_). The projection of the corresponding larger time-averaged amplitude and less tilted magnetic dipole quickly turns in the x–y plane (m_x,y_) in the 0° azimuthal orientation, whereas the smaller time-averaged amplitude and more tilted magnetic dipole slowly rotates in the 90° azimuthal orientation (see Supplementary Information Fig. S4 and b-d segments in Supplementary Videos S1 and S2).

At both representative extrema (negative index: 740 nm; maximal handedness dependence: 680 nm) identified in the case of illumination by circularly polarized light, the multipoles rotate on the nanoring and nanocrescents in each layer; however, the relative phase on the nanoring and satellite nanocrescents is layer-specific (Figs. 5d, 6d, Supplementary Videos S3, S4, see Supplementary Information Fig. S3 and Supplementary Videos S5, S6).

The peculiarity of the negative index location is that dipoles are excited on all composing 3D nano-objects, all of them originate from lateral charge separation, except on the concave nanoring and on the convex nanocrescents in the upper convex layer, which support twisted and tilted charge separation, respectively (see a segments in Supplementary Videos S3 and S4). The inspection of the time-evolution shows that in the lower convex layer, the dipole on the nanoring is perpendicular to the dipoles on the nanocrescents, whereas all dipoles are synchronous on the nanocrescents.

In the concave layer, the relation of dipoles on the ring and nanocrescents varies between perpendicular and synchronous-parallel on the lower surface and between perpendicular and reversal-parallel on the upper surface. As a result, the charge distribution on the concave nanoring becomes a twisted dipole, whereas on the concave nanocrescents it remains a lateral, predominantly horizontal dipole, considering its azimuthal orientation dependent strength.

In the upper convex layer, the charge on the left and right surrounding nanocrescents is in-phase with the charge on the neighbouring half-side of the nanoring, thereby resulting in an extended dipole distributed on the complete convex miniarray. The dipoles on the nanocrescents are reversal on the lower convex—middle concave nano-object pairs, almost in-phase on the nanocrescents but twisted (by approximately 106°) on the opposing surfaces of the nanoring in the middle concave layer, and then again are reversal on the middle concave—upper convex nano-object pairs for both handednesses (see a segments Supplementary Video S3, S4).

The twisted-rotating dipoles on the concave nanoring surfaces result in the appearance of a rotating-tilted electric dipole in 3D, which manifests itself in the continuously-rotating slightly-tilted closed loop of the **D** displacement vector that is observable in a time-varying vertical plane (e.g. x–z and y–z planes: D _xz_, D_yz_).

These are accompanied by **H**-field vortices in the plane orthogonal to the rotating **D** loop (e.g. y–z and x–z planes: corresponding H_yz_, H _xz_) and by the projection of a continuously rotating slightly-tilted largest time-averaged amplitude magnetic dipole observable in the x–y plane (m_x,y_). All phenomena exhibit intermediate characteristics compared to the two orthogonal azimuthal orientations in the case of linearly polarized light illumination (see Supplementary Information Fig. S4 and b–d segments in Supplementary Videos S3 and S4).

The peculiarity of the spectral location, where handedness sensitivity is the highest, is that predominantly quadrupoles are excited on all composing 3D nano-objects. Exceptions are the concave nanocrescents that support dipoles originating from lateral charge separation, and the convex nanocrescents in the upper layer that support tilted dipolar charge separation as well as weak quadrupoles in a noticeable fraction of each cycles (see Supplementary Information Fig. S3 and a segments in Supplementary Videos S5, S6, provided as a reference).

In the absence of the tilted and twisted electric dipole on the concave nanoring, there are no closed loops in the **D** displacement vector in the vertical planes, and no well-defined **H** vortices appear in the orthogonal planes, and a corotating magnetic dipole is not observable on the nanoring (see b–d segments Supplementary Video S5 and S6).

## Discussion

In both cases of linear and circular polarized light illumination copolarized transmittance is dominant and exhibits azimuthal orientation and handedness dependence in either the single-layers or the multilayer. On the multilayer, the location of the maximal difference beween transmittance in the two azimuthal orientations is forward shifted, whereas the maximal handedness difference appears at the same location, as on the single convex layer.

Both the linear and circularly polarized light illumination result in cross-polarized transmittance signal components that do not show azimuthal orientation and handedness dependence in either single-layers or the multilayer. Asymmetric transmission occurs in extended bands for both types of the copolarized signals, namely for illumination with linearly and circularly polarized light as well, when it is transmitted through the single layer convex pattern. Asymmetric transmission occurs for the copolarized transmittance at the same spectral locations, when the convex layer is illuminated with forward and backward propagating linearly polarized light in 90° azimuthal orientation and with forward/backward propagating circularly light of right/left-handedness. The asymmetric dichroism accumulates for circularly polarized light illumination, when convex–concave–convex layers are stacked to compose a multilayer.

When the transmitted signal components are computed based on the linear base representation of the Jones transmission matrix elements, the approached zero-crossing of the difference between the copolarized transmittances of left and right handed circularly polarized light makes it possible to predict the location of the NIM phenomenon.

A well-defined negative index appears in the 0° azimuthal orientation in a wavelength interval, where an LSPR supported by convex nano-objects is coupled efficiently with those on the concave layer. Moreover, this mode is capable of decoupling the SPP mode from U resonance, which arises on the concave nano-objects in the case of linearly polarized light illumination in the 90° azimuthal orientation, as well as from the LSPR, which arises in the case of circularly polarized light illumination.

At the NIM phenomenon-related extrema arising in case of illumination by linearly polarized light predominantly dipolar charge distribution develops on the nano-objects composing the miniarray in the multilayer. Quadrupoles appear intermittently on the central nanoring in the lower convex and middle concave layers, when illumination by linearly polarized light is realized in either the 0° or 90° azimuthal orientations. In the 0° azimuthal orientation a strong rotating-tilted electric dipole appears on the concave nanoring caused be the rotation (turning) of the electric dipole on the lower (upper) surface. This results in a rotating **D** displacement vector loop accompanied by **H**-field vortices, which are observable in orthogonal vertical planes, and in an intermediate time-averaged amplitude quickly-turning intermediately-tilted magnetic dipole, which projection is observable in the horizontal plane. In the 90° azimuthal orientation the asynchronous turning (rotation) of the dipoles on the lower (upper) concave nanoring surfaces allows for the smallest time-averaged amplitude slowly-rotating and mostly-tilted magnetic dipole and the less pronounced NIM phenomenon. The intermittent charge distribution consists of quadrupoles on the nanocrescents in the lower convex layer and tilted dipoles on the nanocrescents in the upper convex layer, as well as SPPs on the concave layer in the case of linearly polarized light illumination in the 90° azimuthal orientation.

Twisted dipoles arise on the middle concave rings in the case of circularly polarized light illumination of either handedness, these are accompanied by magnetic dipoles that are moderately advantageous and allow for an intermediate NIM, despite that they continuously rotate and possess the largest time-averaged amplitude and the smallest tilting. In contrast, at the location of maximal handedness sensitivity, the quadrupolar charge distribution is predominant on almost all nano-objects. Based on the time-dependent charge distribution features, besides the synchronized dipoles on the 3D nano-objects, the strong rotating-tilted electric dipole accompanied by a large time-averaged amplitude, more continuously existing and slightly-tilted magnetic dipoles promote the NIM phenomenon. Considering the negative index appearance at the visible region boundary, the application of these complex structures in chiral metamaterial design and NIM phenomenon achievement is proposed.

## Supplementary Information

Supplementary Information.

## Data Availability

The datasets used and/or analysed during the current study can be obtained from the corresponding author upon reasonable request.
