# Supplementary material for: Metamaterial properties of Babinet complementary complex structures
Source: Sci Rep. 2023 Mar 22;13:4701. doi: 10.1038/s41598-023-31685-7 (PMC10033689; doi:10.1038/s41598-023-31685-7)
Supplement: Supplementary file 1 — Supplementary Information. [file 41598_2023_31685_MOESM1_ESM.zip › SREP-23-00542-s0.pdf]

Metamaterial properties of Babinet complementary complex structures
Supplementary Information

Emese Tóth[1], Balázs Bánhelyi[2], Olivér Fekete[1], Mária Csete[1]

[1]Department of Optics and Quantum Electronics, University of Szeged, Dóm tér 9, Szeged 6720, Hungary

[2]Department of Computational Optimization, University of Szeged, Árpád tér 2, Szeged 6720, Hungary

✉ mcsete@physx.u-szeged.hu

Optical responses extracted via polarization unspecific power-outflow read-out.

The complete complementary optical responses were extracted polarization unspecifically as well, by realizing power-outflow read-out in the RF module of COMSOL Multiphysics, when single convex and concave layers and multilayers of complex patterns were illuminated with linearly and circularly polarized light (Figs. S1a-d, S2a-d).

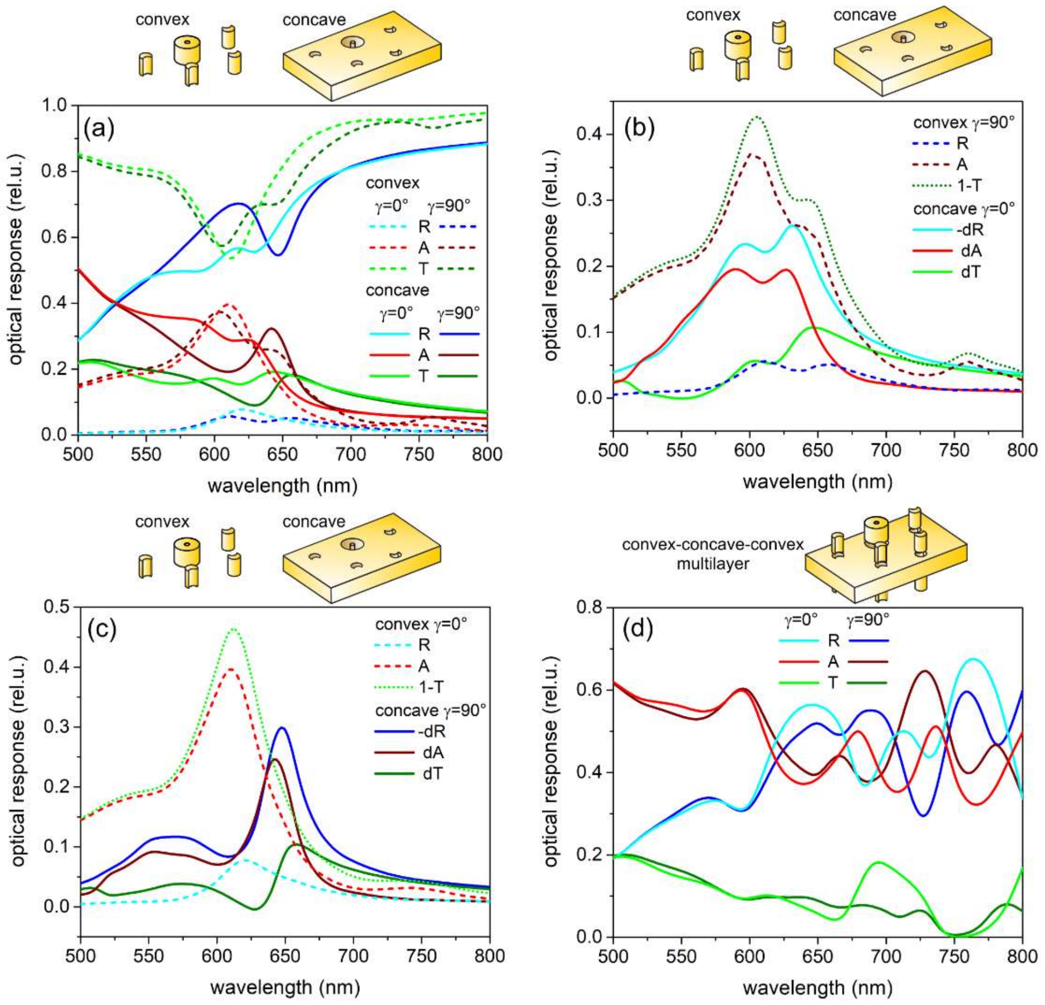

Figure S1 Optical responses extracted via polarization unspecific power-outflow read-out. Absorptance, reflectance, transmittance of (a) single convex and concave layer, (d) convex–concave–convex multilayer, illuminated with linearly polarized light in the 0° and 90° azimuthal orientations. Complementary signals in complementary (b) U-orientation and (c) C-orientation of the complementary convex and concave single layers.

To compare the complementary responses of single layers, the convex transmittance is subtracted from unity, the concave signals are rectified (namely, the signals of a continuous gold film is subtracted), and the rectified concave reflectance is inverted to facilitate comparison of all signal pairs of the convex and concave patterns, as in our previous works (Fig. S1b, c and Fig. S2b, c) (references [44-46] in the manuscript).

When single convex/concave layers are illuminated with linearly polarized light in the 0°/90° azimuthal orientation (U-orientation), the convex nano-objects result in single U resonance-related peak, whereas coupled U resonance and propagating SPP related double peaks appear on the concave single layer (Fig. S1a, b). The illumination of single convex/concave layer with linearly polarized light in the 90°/0° azimuthal orientation (C-orientation) results in double peaks of different origin: the first peak originates from the ring resonance coincident with the mixed C2-C1 resonance on the nanocrescents, whereas the second peak originates from the C1 resonance on the nanocrescents (Fig. S1a, c).

When the optimized multilayer is illuminated with linearly polarized light, in addition to the fingerprint of the resonances supported by the composing layers, novel single/double extrema appear in the 0°/90° azimuthal orientation close to the visible region boundary (Fig. S1d). A remarkable difference is that a shoulder/local maximum is observable at 740 nm/730 nm in the transmittance in the 0°/90° azimuthal orientation.

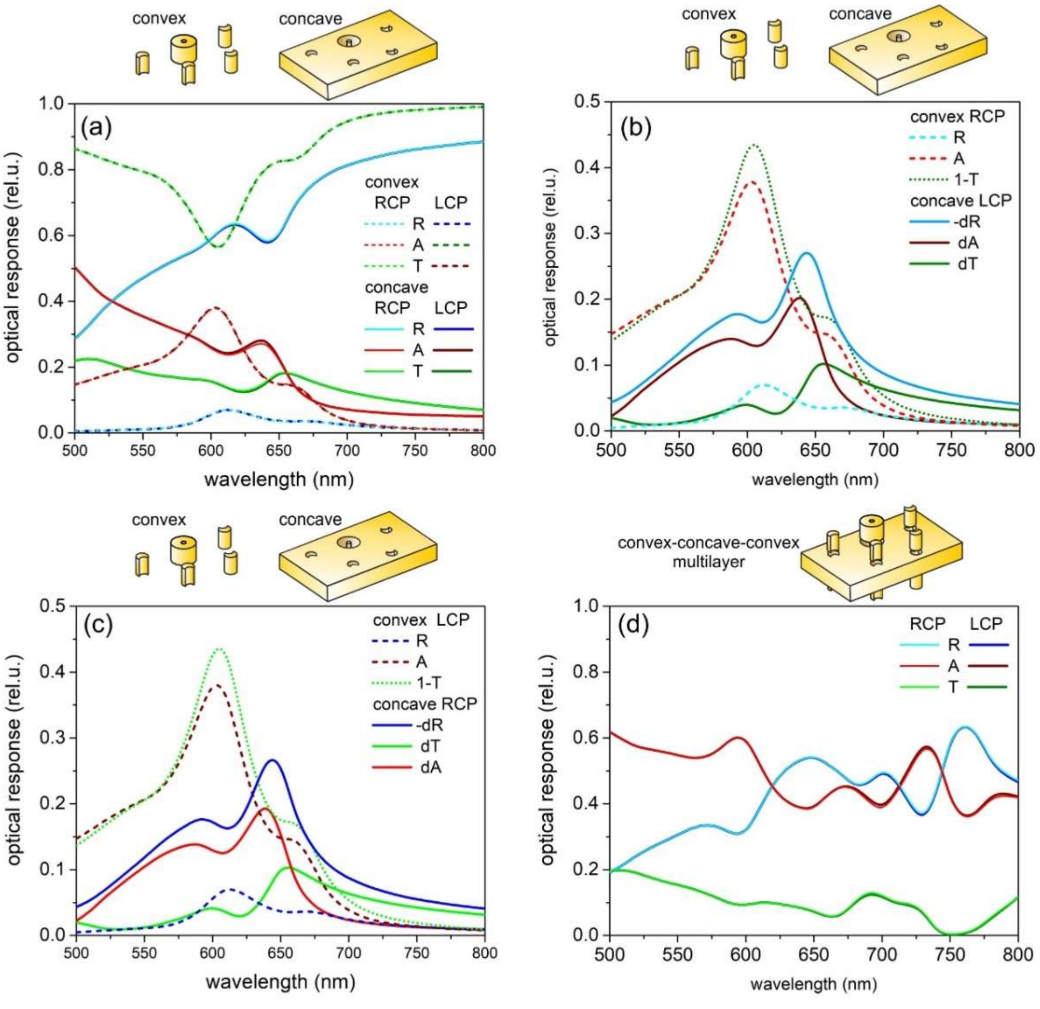

Figure S2 Optical responses extracted via polarization unspecific power-outflow read-out. Absorptance, reflectance, transmittance of (a) single convex and concave layer, (d) convex–concave–convex multilayer, illuminated with circularly polarized light of right (RCP) and left (LCP) handedness. Complementary signals in azimuthal orientations corresponding to the complementary (b) U-orientation and (c) C-orientation of the complementary convex and concave single layers in the case of illumination by linearly polarized light.

When single layers are illuminated with circularly polarized light of either handedness, both on the convex and concave patterns double peaks appear, which originate from the averaged effect of the coupled U resonance and the ring resonance coincident with the mixed C2-C1 resonances, as well as of the coupled SPP and the C1 resonance, which are spectrally overlapping in couples (Fig. S2a, b, c).
However, the illumination of the complementary single layers with circularly polarized light does not result in completely complementary optical responses, since the global-local maxima in the spectra of the convex patterns overlap with the local-global maxima in the spectra of the concave patterns (Fig. S2b, c).
When the optimized multilayer is illuminated with circularly polarized light, in addition to the fingerprints of the resonances supported by composing layers, novel single (double) extrema appear in transmittance and reflectance (absorptance) close to the visible region boundary. However, the polarization unspecifically read out transmittance exhibits a coincident shoulder (730 nm) for both handednesses (Fig. S2d).

Inspection of the charge distribution on three-dimensional structures at the reference spectral location.

The inspection of the time-evolution shows that in the lower convex layer, the quadrupole on the nanoring rotates by preserving both the lateral and vertical charge distributions. Twisted quadrupoles evolve on the nanocrescents, that are created by the asynchronously rotating charges on their lower and upper surfaces.

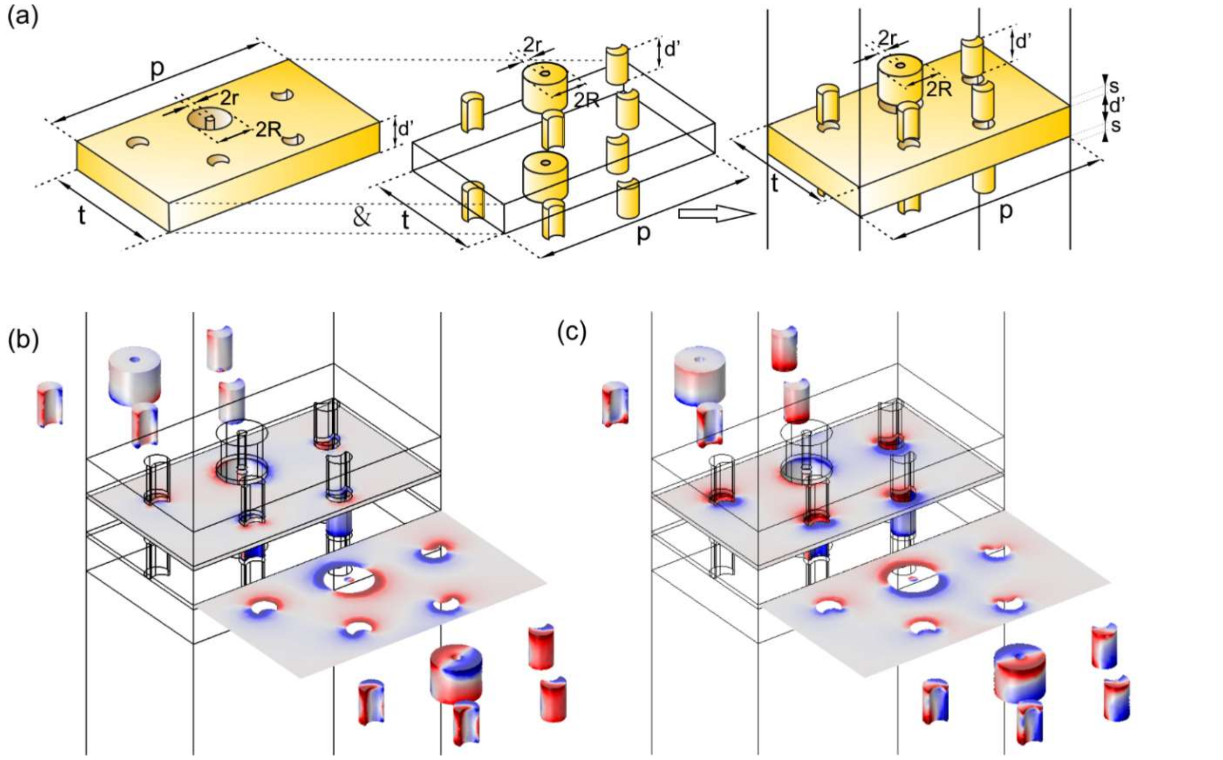

Figure S3 Inspection of the charge distribution on the three-dimensional multilayers. (a) Schematic drawing of the three-dimensional multilayer constructed with the consecutive convex–concave–convex pattern-layers. Charge distribution provided as a reference at the maximal handedness dependence (680 nm) on the multilayer in case of (b) right-handed and (c) left-handed circularly polarized light illumination (see Supplementary Videos S5 and S6 online).

In the concave layer the relative orientation of the surface dipoles on the nanoring and on the nanocrescents varies, since the charge rotates on the nanoring continuously, but it turns on the nanocrescents cyclically. As a result, the three-dimensional charge distribution on the nanoring becomes quadrupolar, while on the nanocrescents remains predominantly dipolar due to the synchronously varying lateral charge separation on their lower and upper surfaces.

In the upper convex layer, the charge rotates synchronously on the lower and upper surfaces of the nanoring, thereby a quadrupole appears on the central nanoring, as in the other layers. In contrast, the dipolar charge distribution on the nanocrescents turns cyclically with different phase on the opposite surfaces, thus beside the tilted dipolar distribution, quadrupolar charge separation also appears intermittently in three-dimensions.

The dipoles on the nanocrescents are reversal at the interface of the lower convex layer –middle concave nano-object pairs; are in-phase on the nanocrescents but twisted cyclically with 180° on the nanoring on the opposing surfaces of the middle concave layer, thereby creating a quadrupole; then again are reversal at the interface of the middle concave – upper convex nano-object pairs (Fig. S3, and a segments in Supplementary Videos S5, S6).

Caused by the quadrupolar distribution on the concave nanoring the **D** displacement vectors are enhanced only close to the lower surface, which cannot create closed loops, and are not surrounded by **H**-field vortices in the vertical plane cross-sections. On the contrary, signature of magnetic dipoles is recognizable on the nanocrescents in the horizontal plane cross-section (see b-d segments in Supplementary Videos S5 and S6).

Correlation between the negative index and the characteristics of the accompanying magnetic dipoles

The time-dependence of the amplitude and orientation of the $\mathbf{p}_m$ magnetic dipoles corresponding to the **D** displacement current loops that arise on the nano-objects at the spectral location of the NIM phenomenon was also investigated. The **D** displacement current was integrated throughout spherical shells embedding the individual nano-objects, and the corresponding $\mathbf{p}_m$ magnetic dipole was derived as their curl. The amplitude of the magnetic dipole is significantly larger on the nanoring than on the nanocrescents, thereby the magnetic dipole on the miniarray is governed by this dipole in all inspected configurations.

In case of illumination with linearly polarized light the 0° azimuthal orientation (γ, measured from the y axis) is advantageous. This is because both the maximal and time-averaged amplitude of the magnetic dipole arising on the nanoring is larger in this orientation. The charge rotation allows for shorter phase-interval, during which the magnetic dipole orientation is reversed on the nanoring, namely quick turning occurs in the 0° azimuthal orientation. Accordingly, the dipole is predominantly oriented along the x axis ($\alpha=90°-\gamma$, i.e. 0° value is taken on). Both the maximal and average tilting of the magnetic dipole on the nanoring compared to the horizontal plane (qualified by the $\theta=90°+\varphi$ inclination values, φ measured from z axis, $\theta=90°$ corresponds to xy-plane), is considerably smaller. The larger amplitude accompanied by smaller out-of-plane tilting of the more continuously existing magnetic dipole on the nanoring correlates with that the NIM phenomenon is more pronounced in the 0° azimuthal orientation.

In case of illumination with circularly polarized light the maximal and time-averaged amplitude of the magnetic dipole on the nanoring is slightly larger for the right-handedness. The charge rotation is continuous on the nanoring for both handednesses. Both the maximal and average tilting compared to the horizontal plane are slightly larger/smaller for the right/left handedness. These balanced phenomena allow for similar NIM, that is just slightly promoted in the case of right handedness.

The maximal (time-averaged) amplitude of the magnetic dipole on the nanoring achieved via left and right handed circularly polarized light illumination is smaller (larger) compared to the amplitude reached via linearly polarized light illumination either in the 0° or in the 90° azimuthal orientation. The magnetic dipoles are continuously rotating on the nanoring in a plane that is just slightly tilted compared to the horizontal plane. The twisted-rotating electric dipoles and the resulted magnetic dipole related phenomena allow for intermediate NIM, when circularly polarized illumination is used, handednesses independently.

In conclusion, the existence of larger time-averaged amplitude, less out-of-plane tilted, more continuously existing magnetic dipole is more advantageous to achieve a more pronounced NIM.

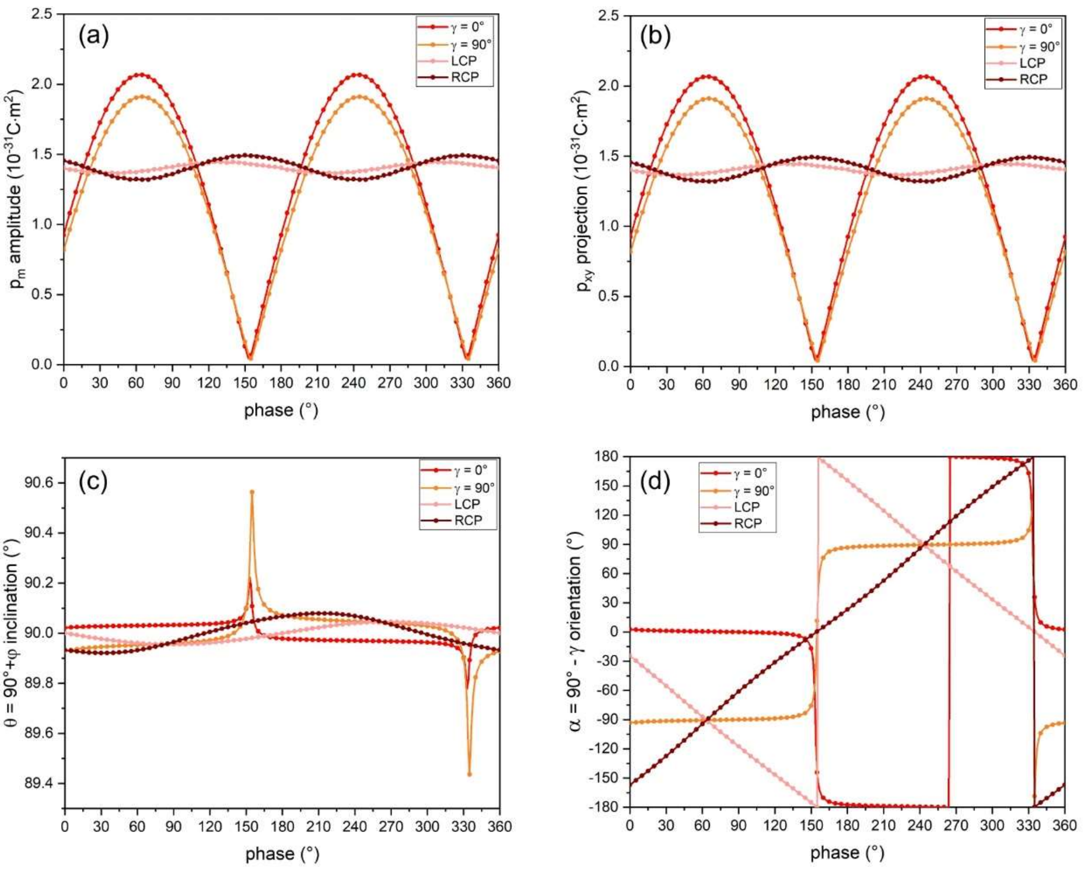

Figure S4 The (a) p_m amplitude, (b) p_{xy} projection, (c) θ inclination (ϕ values measured from the z axis), (d) α azimuthal orientation (γ measured from the y axis) of the $\mathbf{p}_m$ magnetic dipole arising in the case of illumination with (**E**-field oscillation direction in the 0° and 90° azimuthal orientation) linearly and (right and left-handed) circularly polarized light.

Legends:

Figure S1 Optical responses extracted via polarization unspecific power-outflow read-out. Absorptance, reflectance, transmittance of (a) single convex and concave layer, (d) convex–concave–convex multilayer, illuminated with linearly polarized light in the 0° and 90° azimuthal orientations. Complementary signals in complementary (b) U-orientation and (c) C-orientation of the complementary convex and concave single layers.

Figure S2 Optical responses extracted via polarization unspecific power-outflow read-out. Absorptance, reflectance, transmittance of (a) single convex and concave layer, (d) convex–concave–convex multilayer, illuminated with circularly polarized light of right (RCP) and left (LCP) handedness. Complementary signals in azimuthal orientations corresponding to the complementary (b) U-orientation and (c) C-orientation of the complementary convex and concave single layers in the case of illumination by linearly polarized light.

Figure S3 Inspection of the charge distribution on the three-dimensional multilayers. (a) Schematic drawing of the three-dimensional multilayer constructed with the consecutive convex–concave–convex pattern-layers. Charge distribution provided as a reference at the maximal handedness dependence (680 nm) on the multilayer in case of (b) right-handed and (c) left-handed circularly polarized light illumination (see Supplementary Videos S5 and S6 online).

Figure S4 The (a) p_m amplitude, (b) p_{xy} projection, (c) q inclination (f values measured from the z axis), (d) □ azimuthal orientation (g measured from the y axis) of the $\mathbf{p}_m$ magnetic dipole arising in the case of illumination with (**E**-field oscillation direction in the 0° and 90° azimuthal orientation) linearly and (right and left-handed) circularly polarized light.

Video S1. (a) Charge distribution at the spectral location (740 nm) of negative index on the multilayer; well-defined (b) and (c) sub-dominant D_{xz} and super-dominant D_{yz} loops accompanied by H_{yz} and H_{xz} vortices in orthogonal vertical planes; and (d) the projection of the quickly rotating predominantly horizontal magnetic dipole (m_{xy}) on the x-y plane during linearly polarized light illumination in 0° azimuthal orientation.

Video S2. (a) Charge distribution at the spectral location (740 nm) of negative index on the multilayer; well-defined (b) and (c) super-dominant D_{xz} and sub-dominant D_{yz} loops accompanied by H_{yz} and H_{xz} vortices in orthogonal vertical planes; and (d) the projection of the slowly rotating predominantly vertical magnetic dipole (m_{xy}) on the x-y plane during linearly polarized light illumination in 90° azimuthal orientation.

Video S3. (a) Charge distribution at the spectral location (740 nm) of negative index on the multilayer; intermediate (b) and (c) D_{xz} and D_{yz} loops accompanied by H_{yz} and H_{xz} vortices in orthogonal vertical planes; and (d) the projection of the continuously rotating magnetic dipole (m_{xy}) on the x-y plane during illumination with right-handed (RCP: +) circularly polarized light.

Video S4. (a) Charge distribution at the spectral location (740 nm) of negative index on the multilayer: intermediate (b) and (c) D_{xz} and D_{yz} loops accompanied by H_{yz} and H_{xz} vortices in orthogonal vertical planes; and (d) the projection of the continuously rotating magnetic dipole (m_{xy}) on the x-y plane during illumination with left-handed (LCP: -) circularly polarized light.

Video S5. (a) Charge distribution at the spectral location (680 nm) of maximal handedness dependence on the multilayer; (b) and (c) absence of D_{xz} and D_{yz} loops, H_{yz} and H_{xz} vortices in orthogonal vertical planes; and (d) absence of a rotating magnetic dipole (m_{xy}) on the x-y plane during illumination with right-handed (RCP: +) circularly polarized light.

Video S6. (a) Charge distribution at the spectral location (680 nm) of maximal handedness dependence on the multilayer; (b) and (c) absence of D_{xz} and D_{yz} loops, H_{yz} and H_{xz} vortices in orthogonal vertical planes; and (d) absence of a rotating magnetic dipole (m_{xy}) on the x-y plane during illumination with left-handed (LCP: -) circularly polarized light.
