# Supplementary figures and images for: Metamaterial properties of Babinet complementary complex structures
Source: Sci Rep. 2023 Mar 22;13:4701. doi: 10.1038/s41598-023-31685-7 (PMC10033689; doi:10.1038/s41598-023-31685-7)

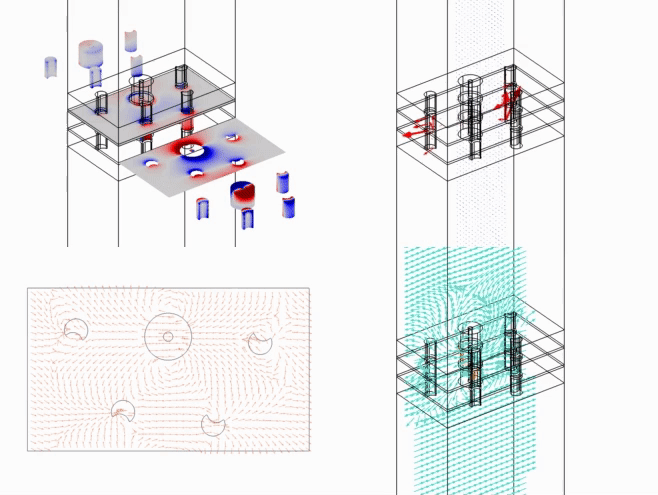

Supplement: Supplementary file 1 — Supplementary Information. [file 41598_2023_31685_MOESM1_ESM.zip › SREP-23-00542-s1.gif]

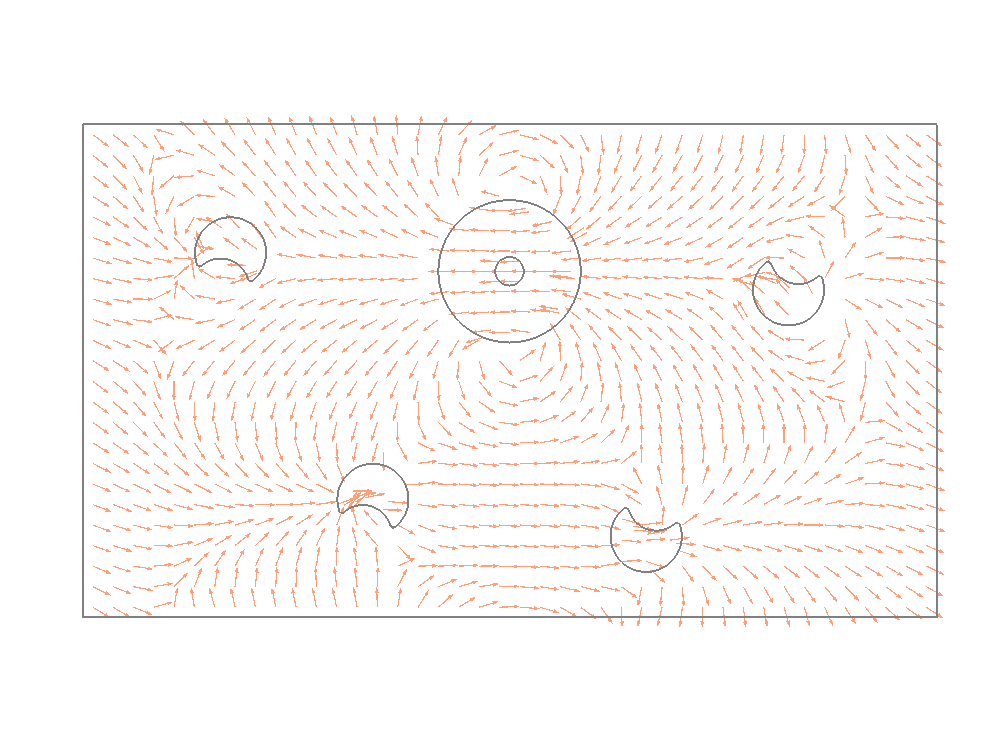

Supplement: Supplementary file 1 — Supplementary Information. [file 41598_2023_31685_MOESM1_ESM.zip › SREP-23-00542-s10.gif]

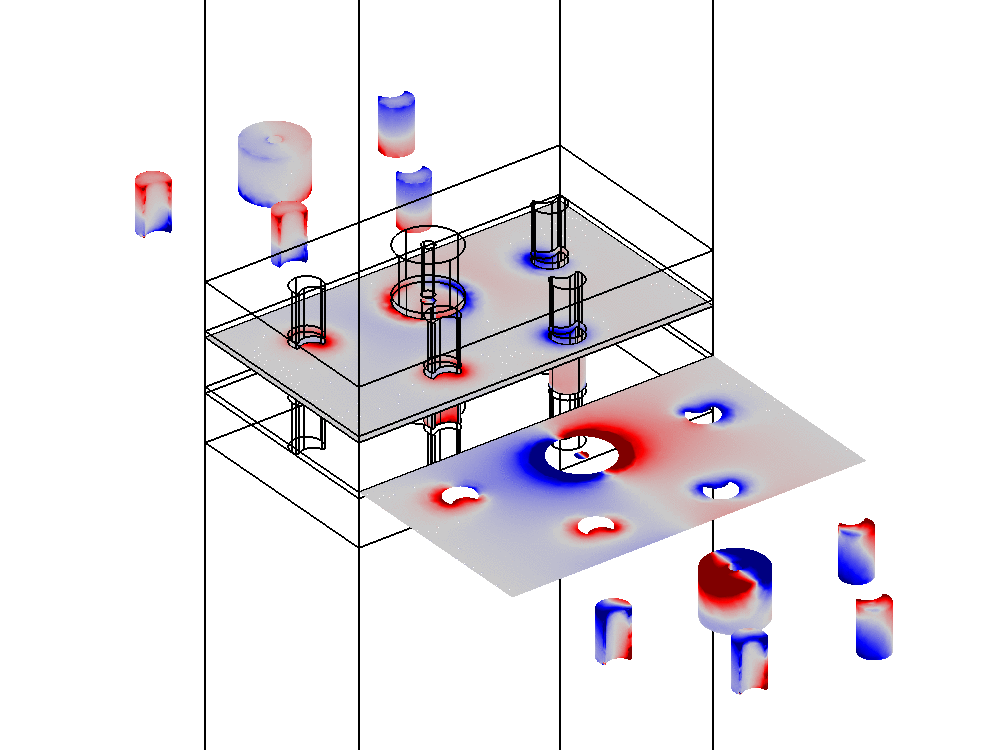

Supplement: Supplementary file 1 — Supplementary Information. [file 41598_2023_31685_MOESM1_ESM.zip › SREP-23-00542-s11.gif]

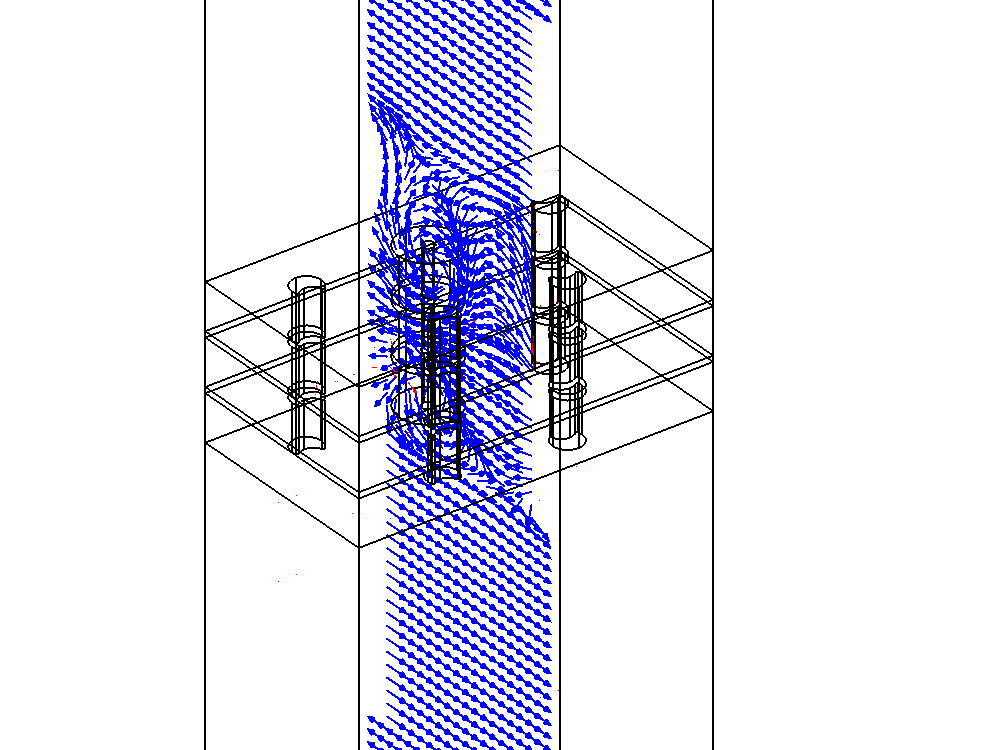

Supplement: Supplementary file 1 — Supplementary Information. [file 41598_2023_31685_MOESM1_ESM.zip › SREP-23-00542-s12.gif]

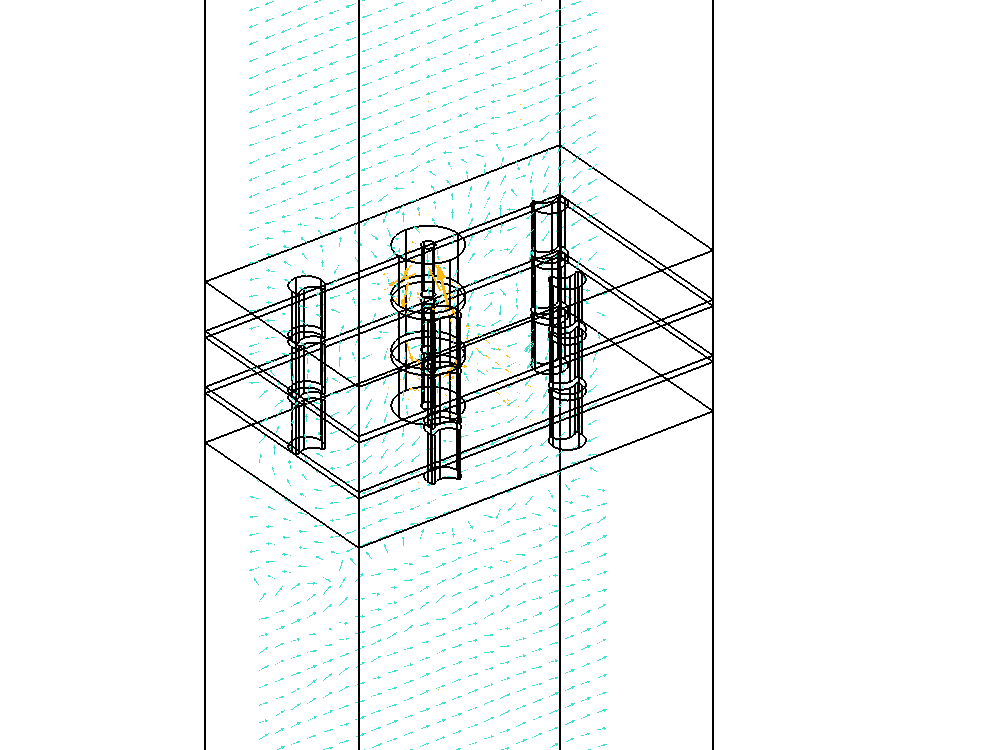

Supplement: Supplementary file 1 — Supplementary Information. [file 41598_2023_31685_MOESM1_ESM.zip › SREP-23-00542-s13.gif]

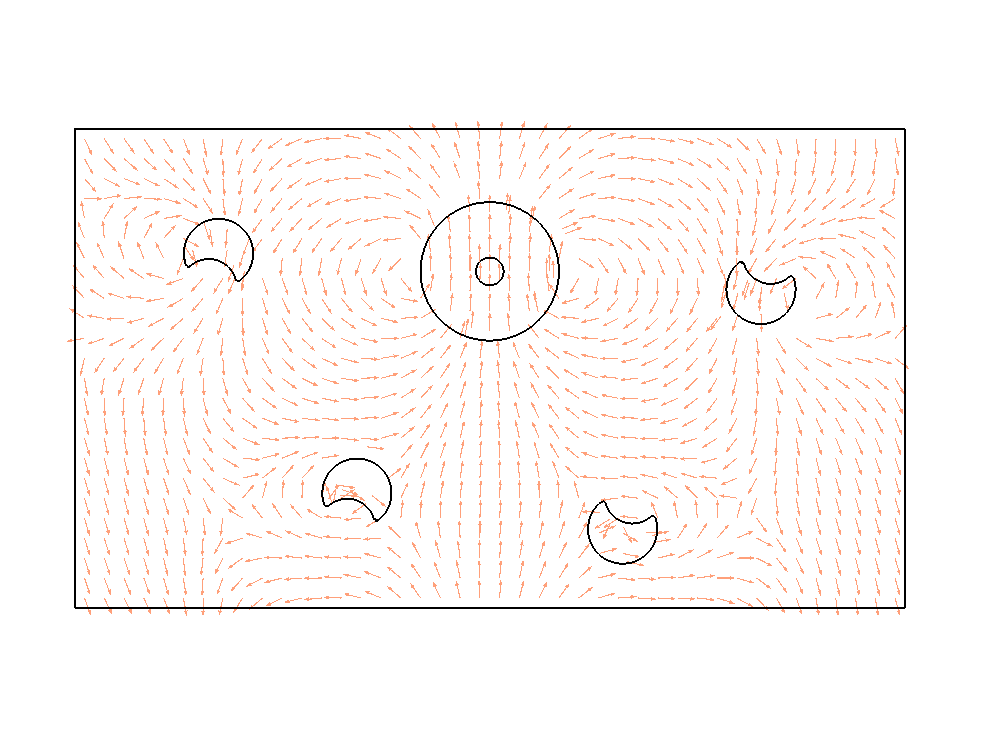

Supplement: Supplementary file 1 — Supplementary Information. [file 41598_2023_31685_MOESM1_ESM.zip › SREP-23-00542-s14.gif]

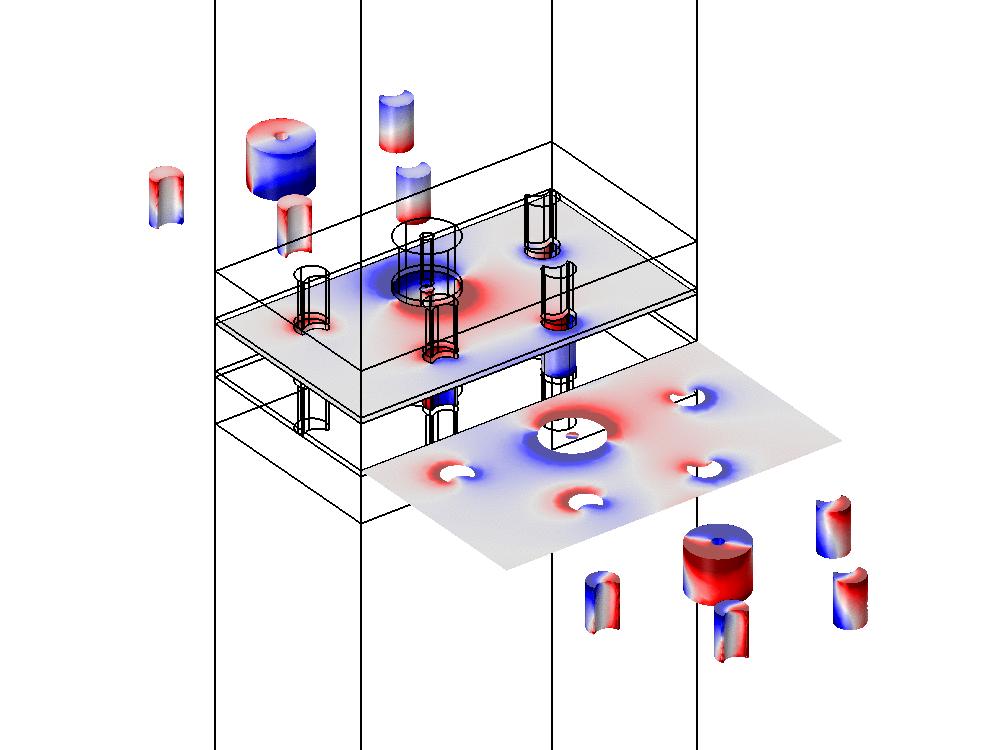

Supplement: Supplementary file 1 — Supplementary Information. [file 41598_2023_31685_MOESM1_ESM.zip › SREP-23-00542-s15.gif]

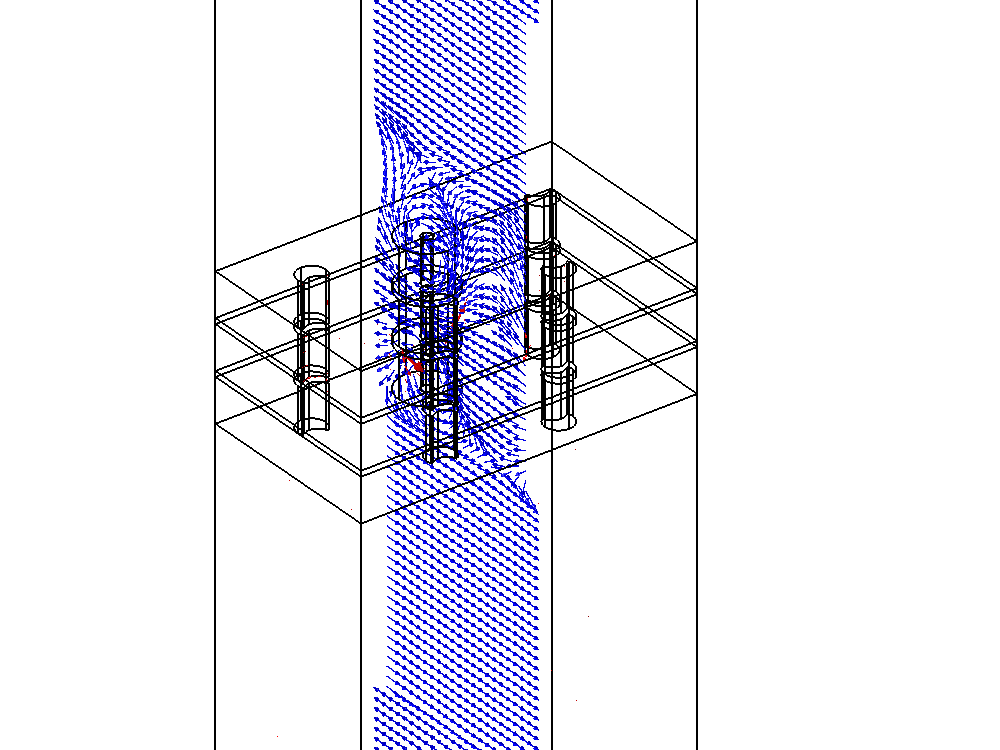

Supplement: Supplementary file 1 — Supplementary Information. [file 41598_2023_31685_MOESM1_ESM.zip › SREP-23-00542-s16.gif]

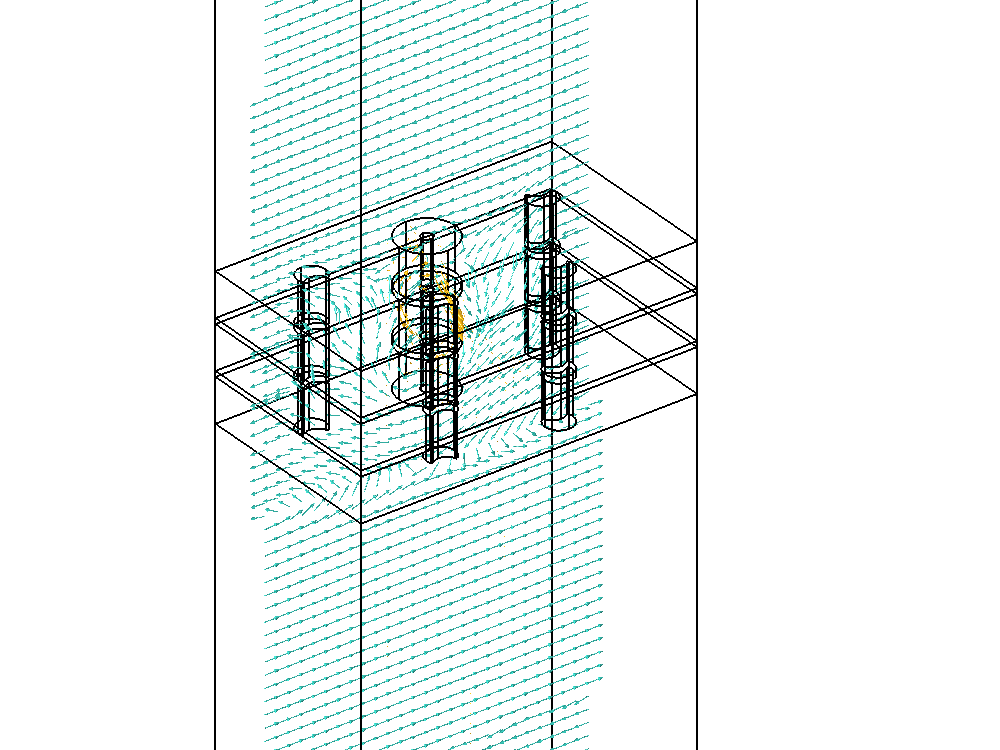

Supplement: Supplementary file 1 — Supplementary Information. [file 41598_2023_31685_MOESM1_ESM.zip › SREP-23-00542-s17.gif]

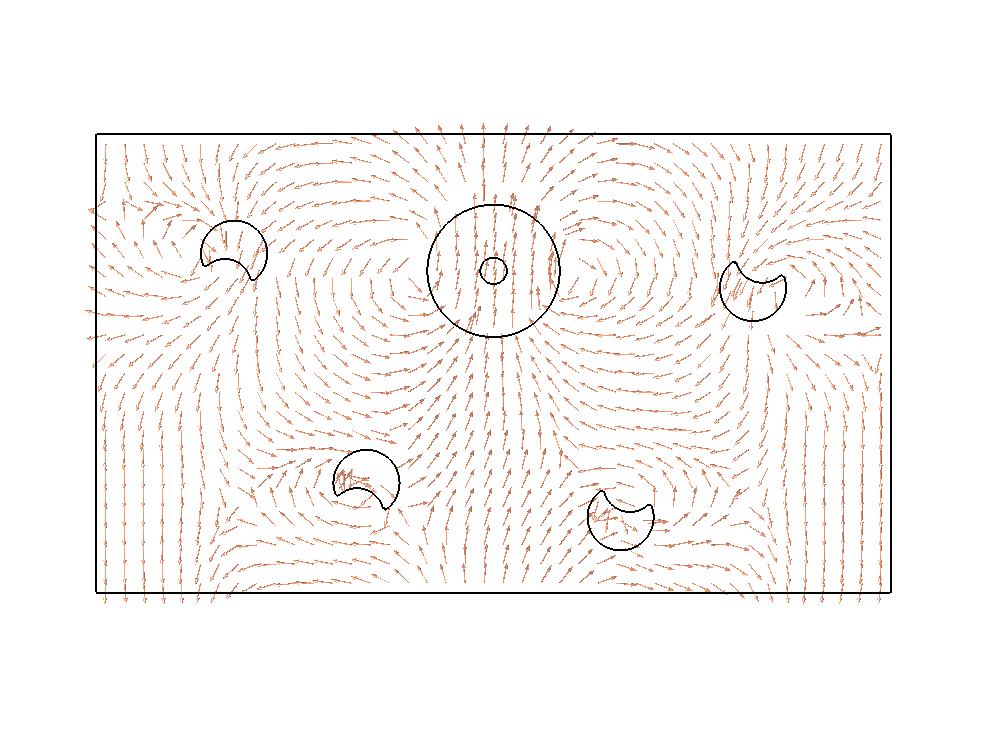

Supplement: Supplementary file 1 — Supplementary Information. [file 41598_2023_31685_MOESM1_ESM.zip › SREP-23-00542-s18.gif]

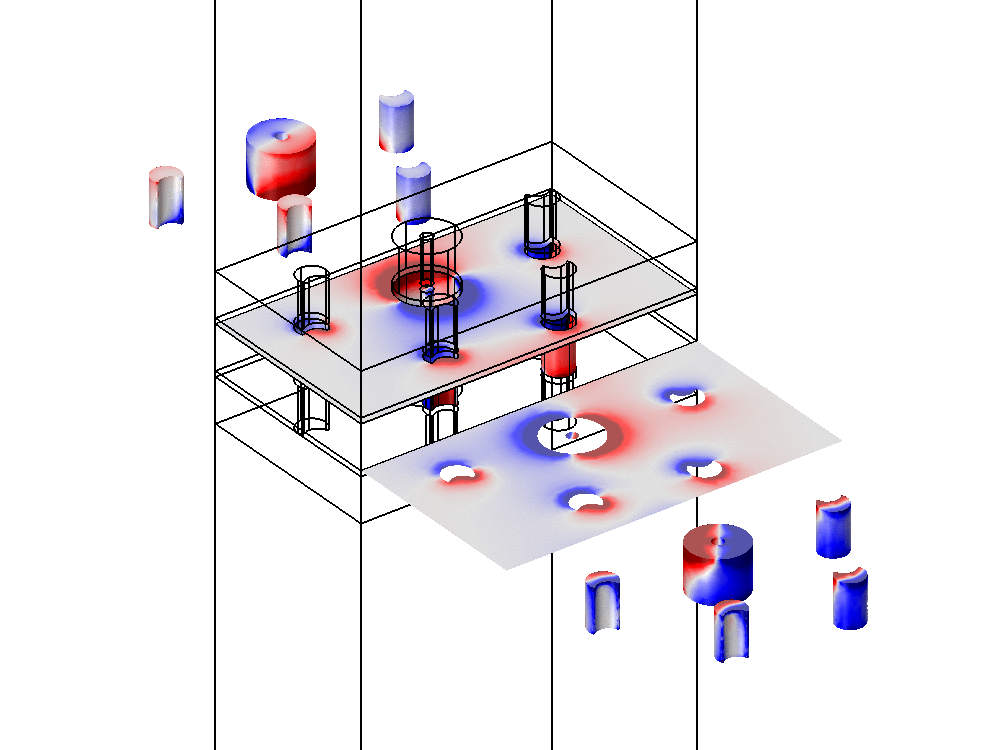

Supplement: Supplementary file 1 — Supplementary Information. [file 41598_2023_31685_MOESM1_ESM.zip › SREP-23-00542-s19.gif]

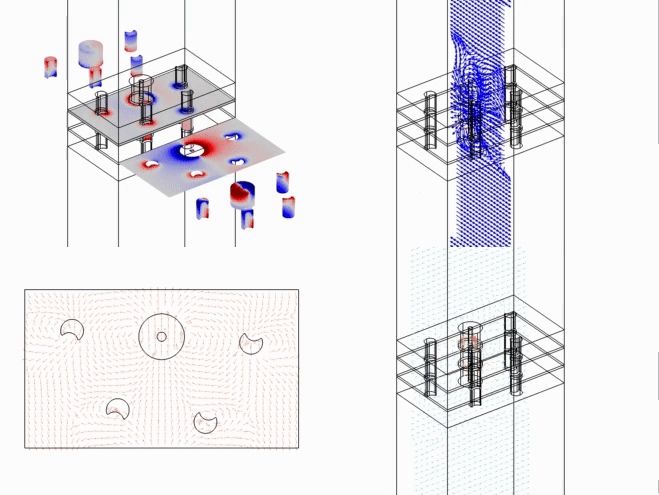

Supplement: Supplementary file 1 — Supplementary Information. [file 41598_2023_31685_MOESM1_ESM.zip › SREP-23-00542-s2.gif]

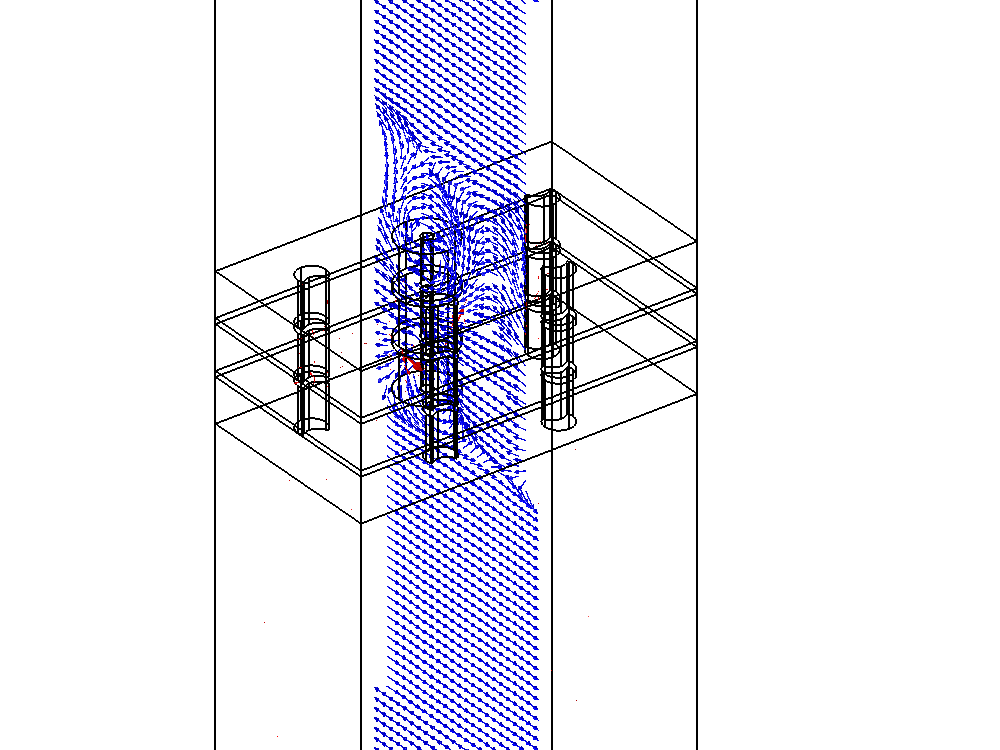

Supplement: Supplementary file 1 — Supplementary Information. [file 41598_2023_31685_MOESM1_ESM.zip › SREP-23-00542-s20.gif]

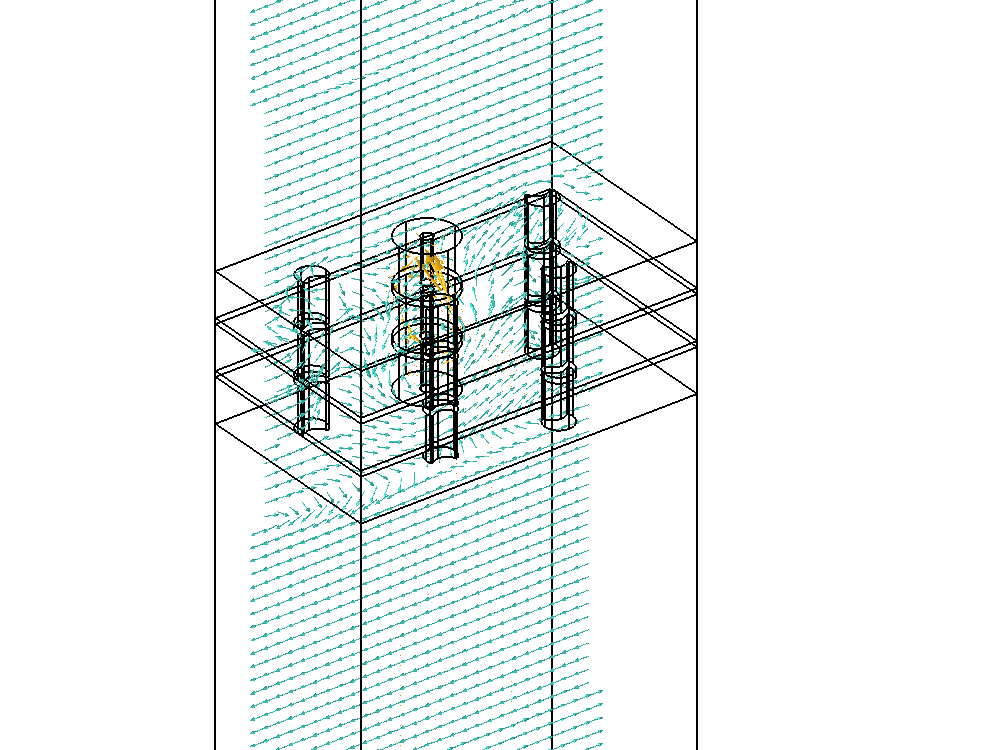

Supplement: Supplementary file 1 — Supplementary Information. [file 41598_2023_31685_MOESM1_ESM.zip › SREP-23-00542-s21.gif]

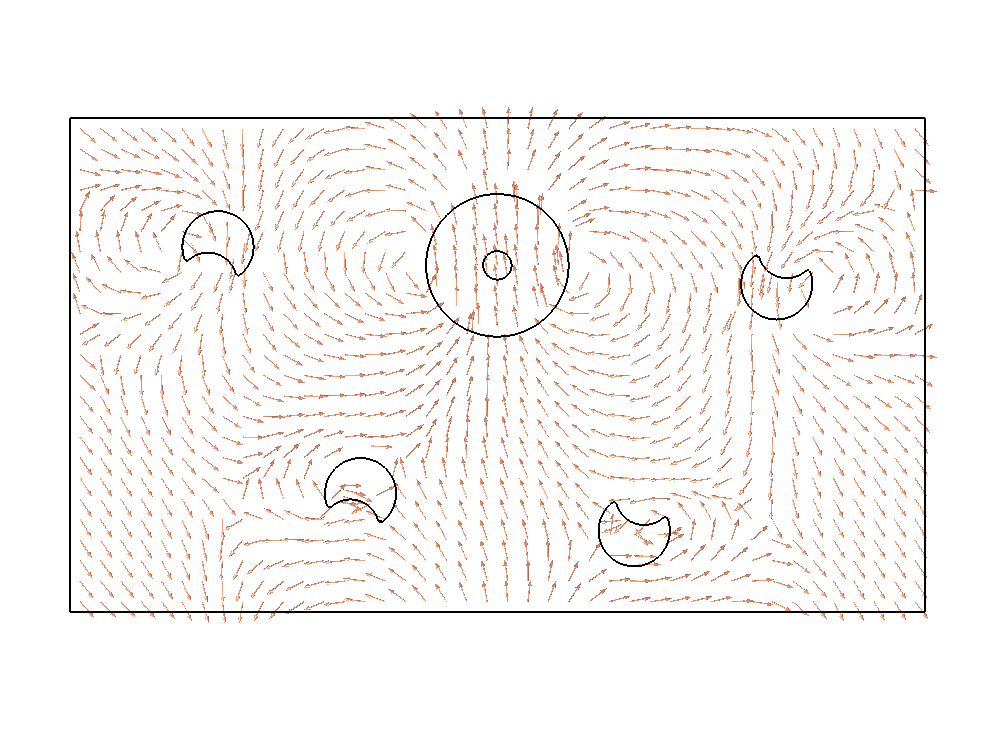

Supplement: Supplementary file 1 — Supplementary Information. [file 41598_2023_31685_MOESM1_ESM.zip › SREP-23-00542-s22.gif]

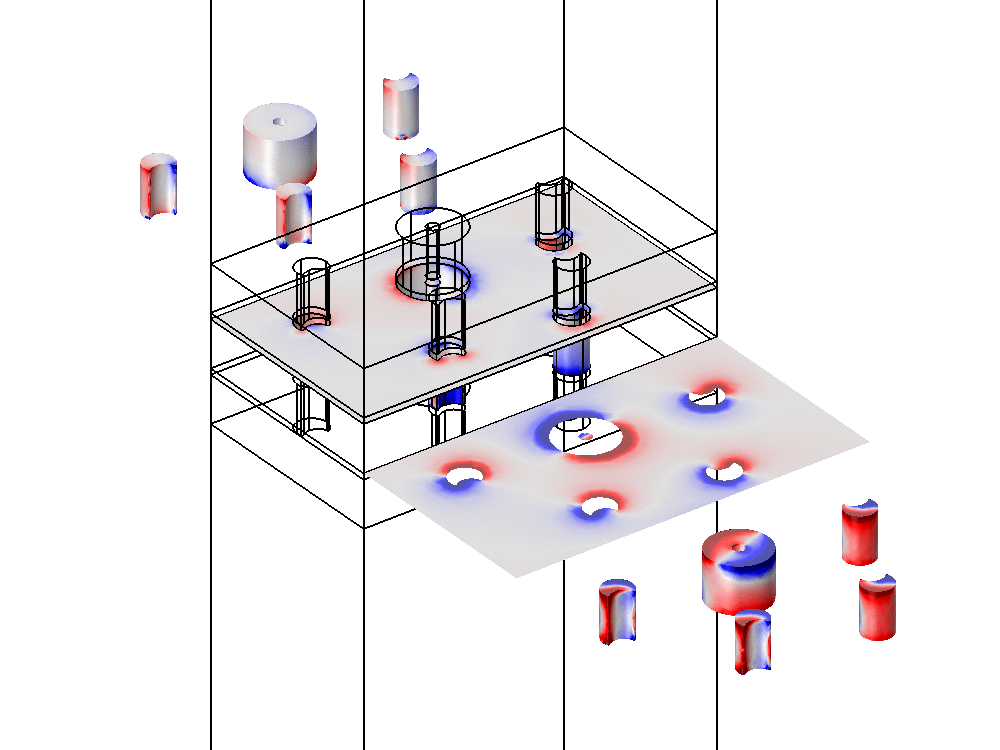

Supplement: Supplementary file 1 — Supplementary Information. [file 41598_2023_31685_MOESM1_ESM.zip › SREP-23-00542-s23.gif]

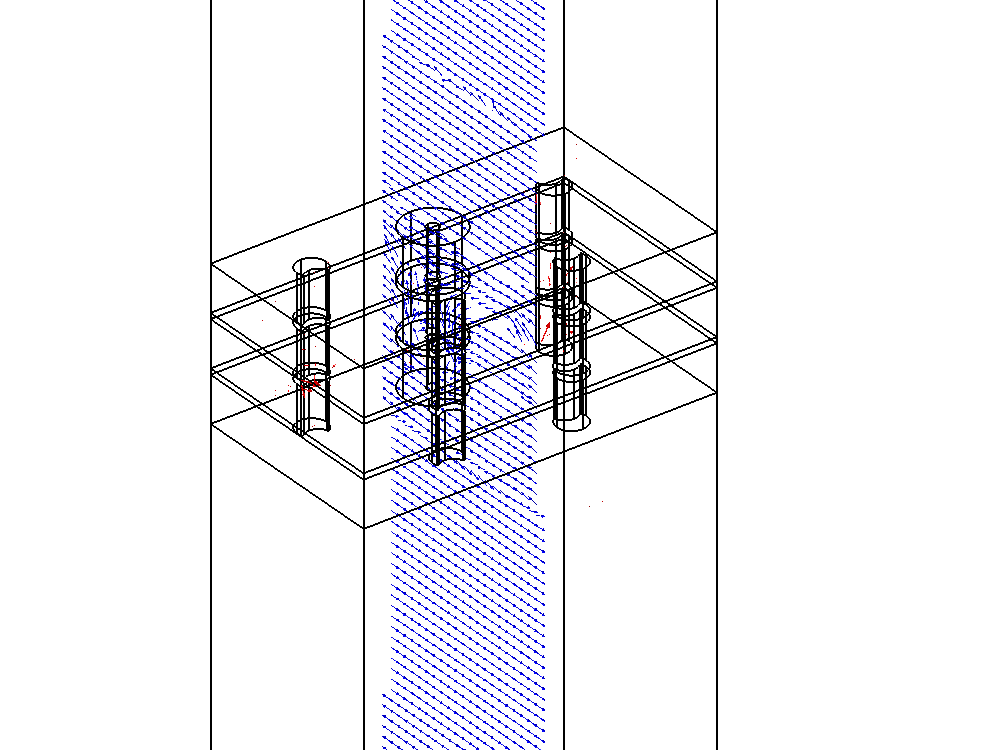

Supplement: Supplementary file 1 — Supplementary Information. [file 41598_2023_31685_MOESM1_ESM.zip › SREP-23-00542-s24.gif]

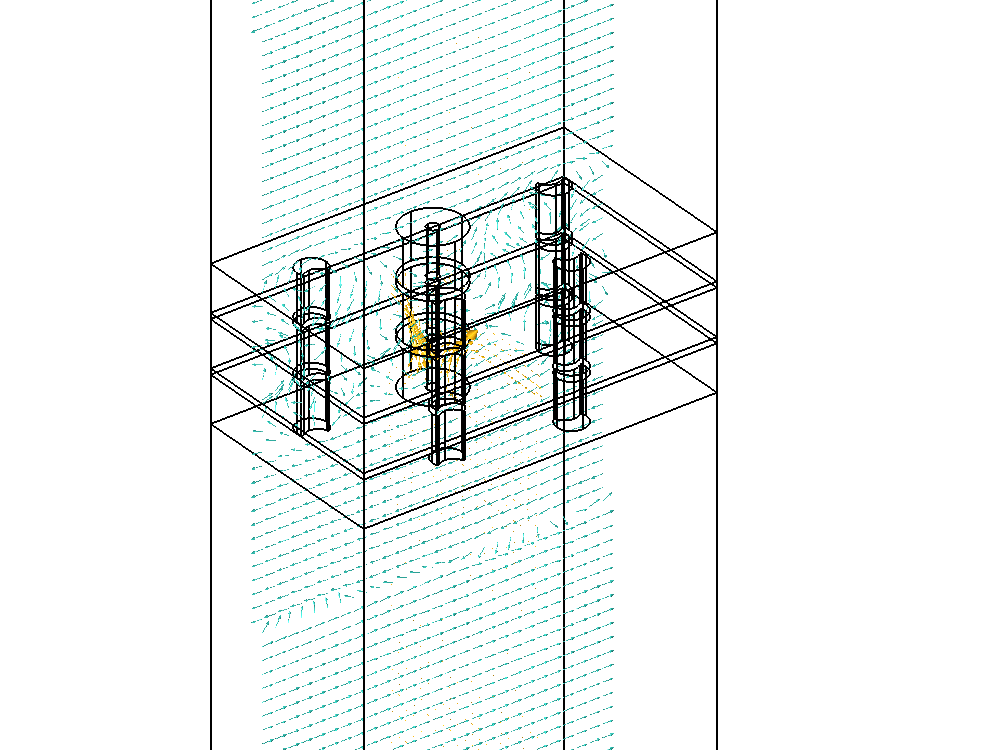

Supplement: Supplementary file 1 — Supplementary Information. [file 41598_2023_31685_MOESM1_ESM.zip › SREP-23-00542-s25.gif]

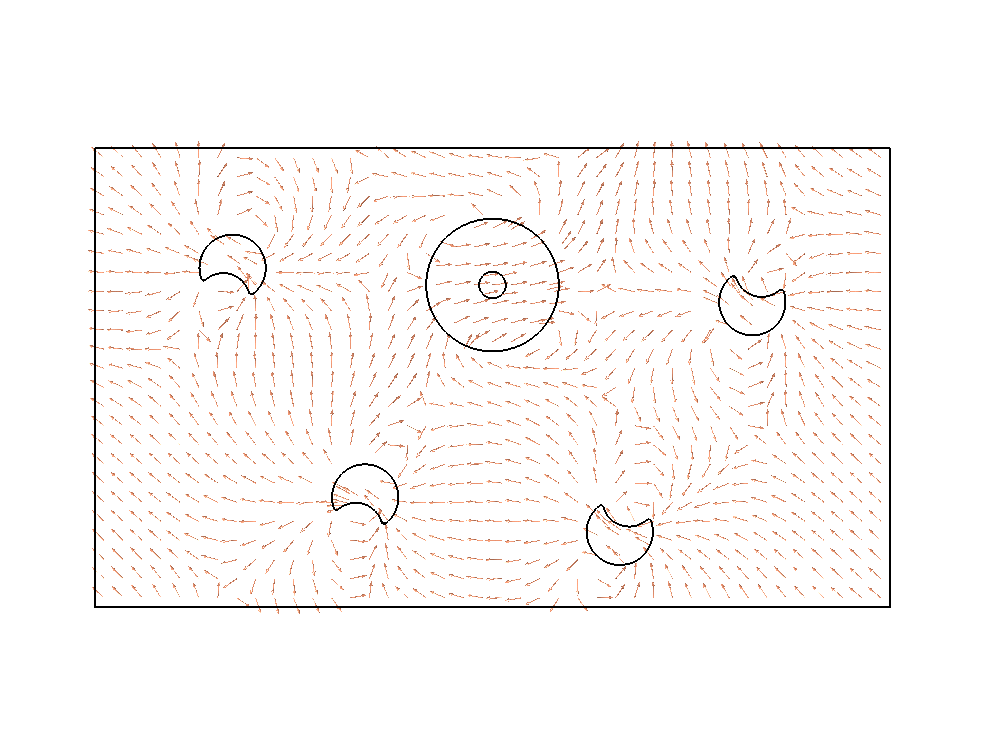

Supplement: Supplementary file 1 — Supplementary Information. [file 41598_2023_31685_MOESM1_ESM.zip › SREP-23-00542-s26.gif]

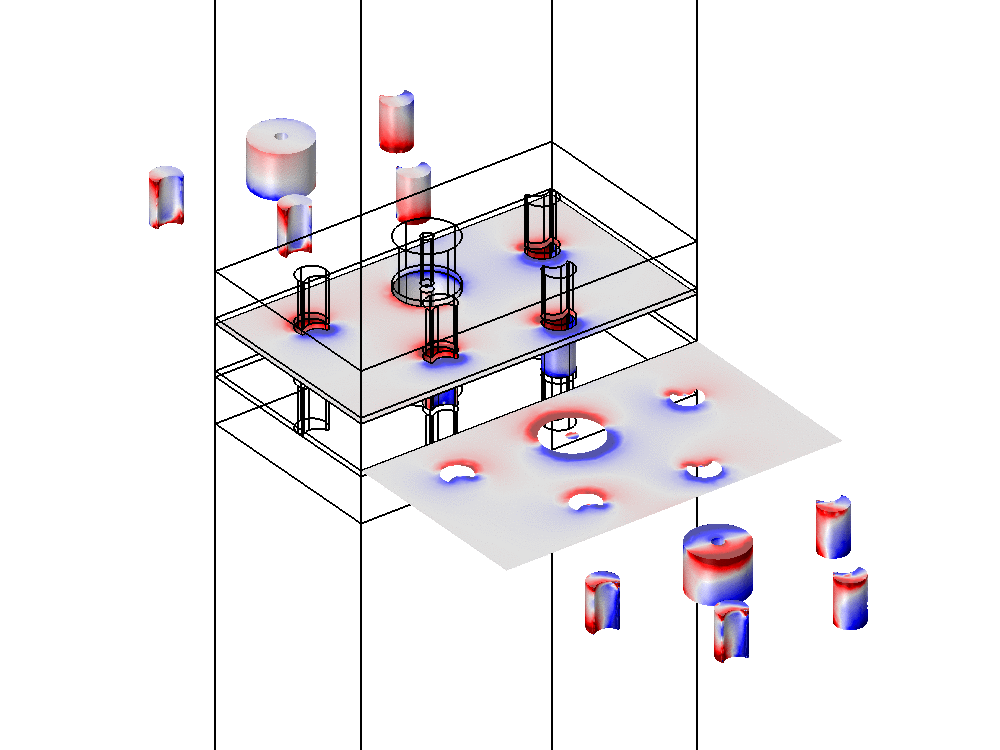

Supplement: Supplementary file 1 — Supplementary Information. [file 41598_2023_31685_MOESM1_ESM.zip › SREP-23-00542-s27.gif]

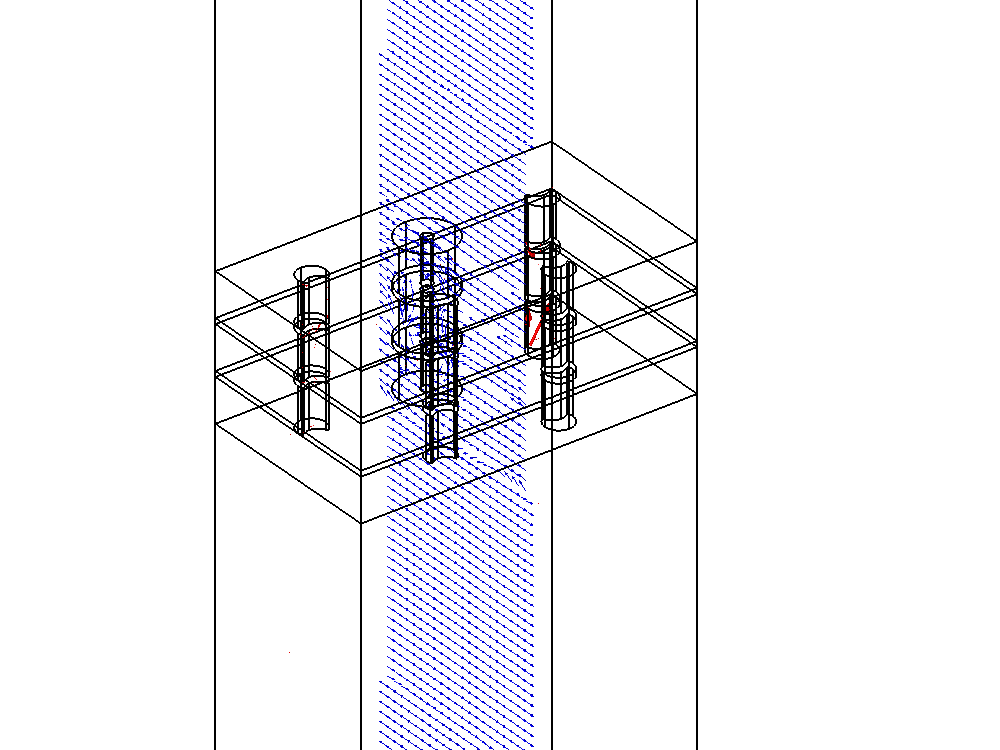

Supplement: Supplementary file 1 — Supplementary Information. [file 41598_2023_31685_MOESM1_ESM.zip › SREP-23-00542-s28.gif]

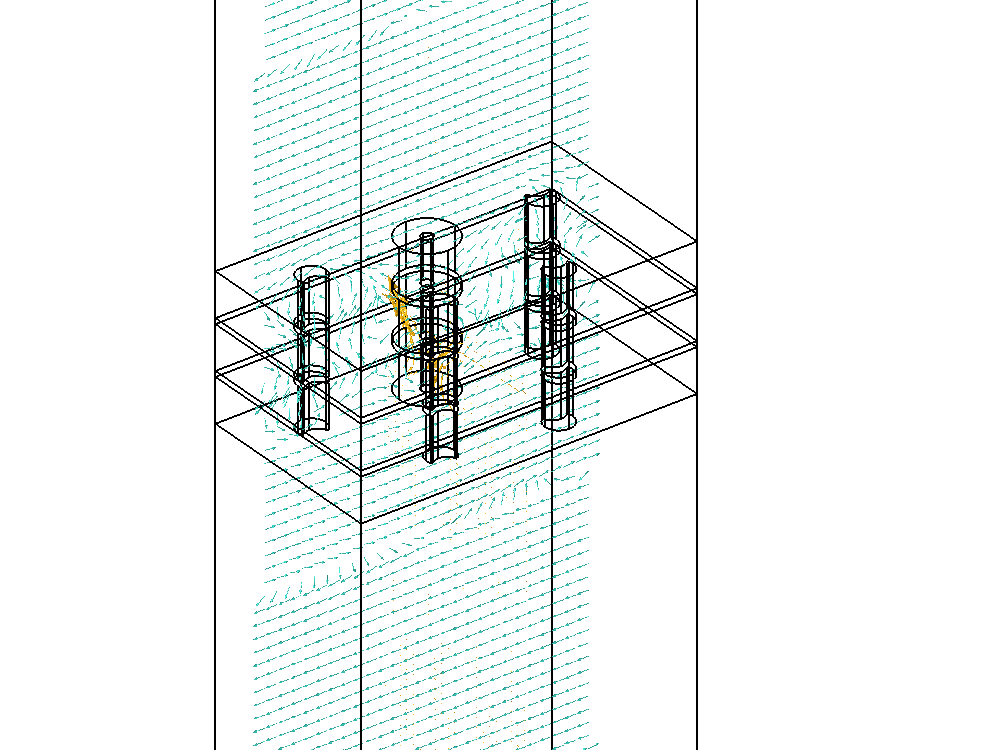

Supplement: Supplementary file 1 — Supplementary Information. [file 41598_2023_31685_MOESM1_ESM.zip › SREP-23-00542-s29.gif]

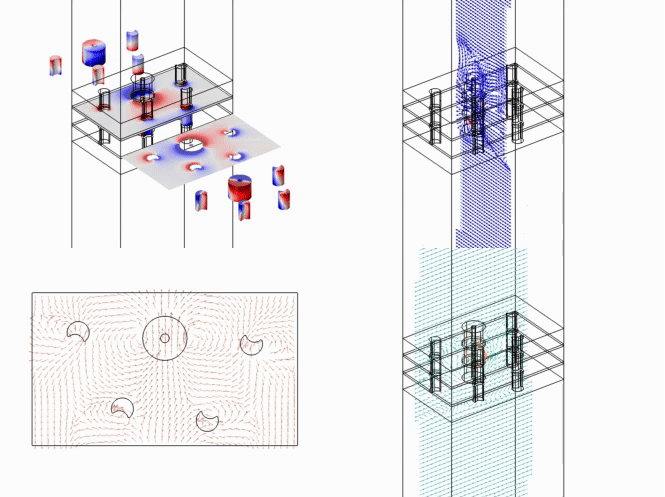

Supplement: Supplementary file 1 — Supplementary Information. [file 41598_2023_31685_MOESM1_ESM.zip › SREP-23-00542-s3.gif]

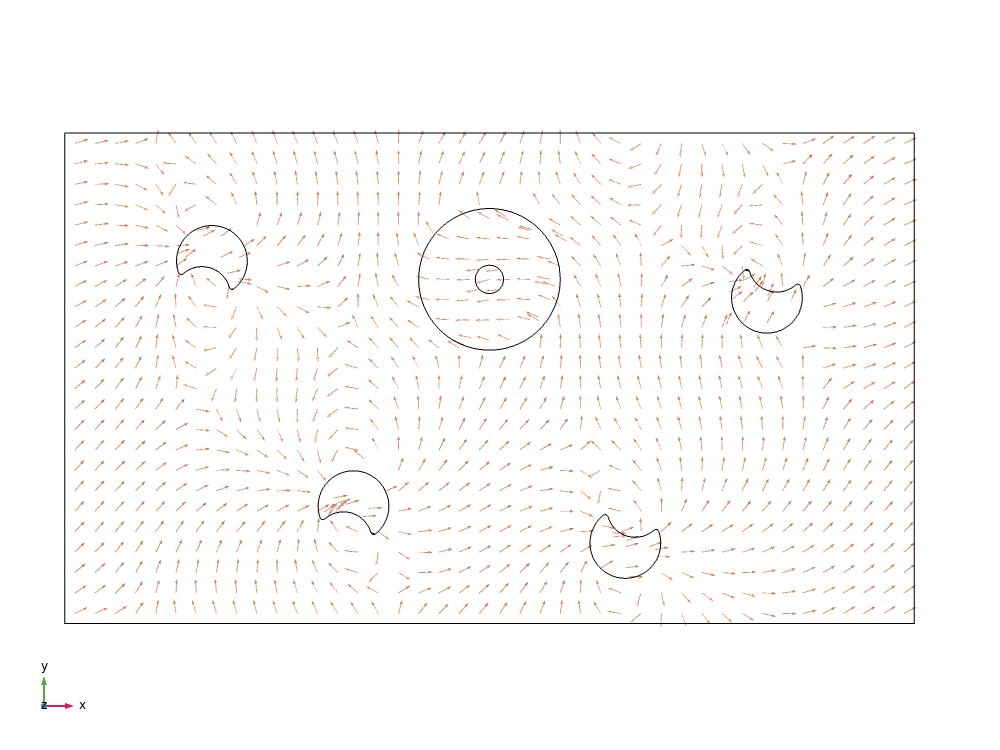

Supplement: Supplementary file 1 — Supplementary Information. [file 41598_2023_31685_MOESM1_ESM.zip › SREP-23-00542-s30.gif]

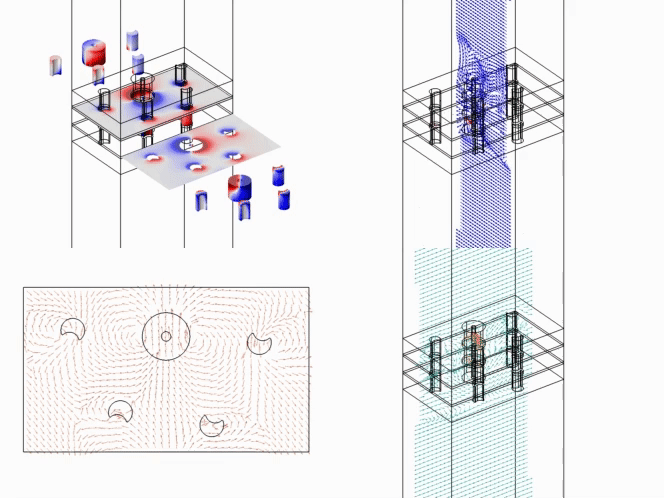

Supplement: Supplementary file 1 — Supplementary Information. [file 41598_2023_31685_MOESM1_ESM.zip › SREP-23-00542-s4.gif]

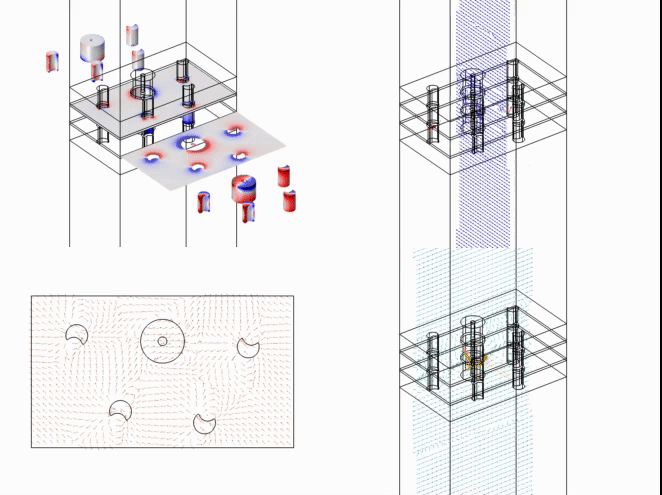

Supplement: Supplementary file 1 — Supplementary Information. [file 41598_2023_31685_MOESM1_ESM.zip › SREP-23-00542-s5.gif]

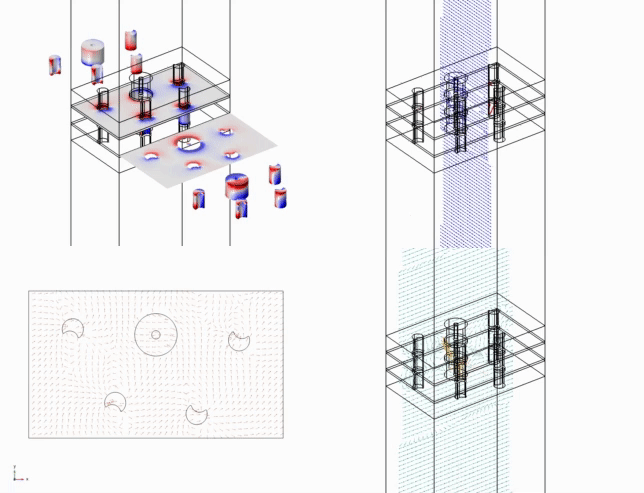

Supplement: Supplementary file 1 — Supplementary Information. [file 41598_2023_31685_MOESM1_ESM.zip › SREP-23-00542-s6.gif]

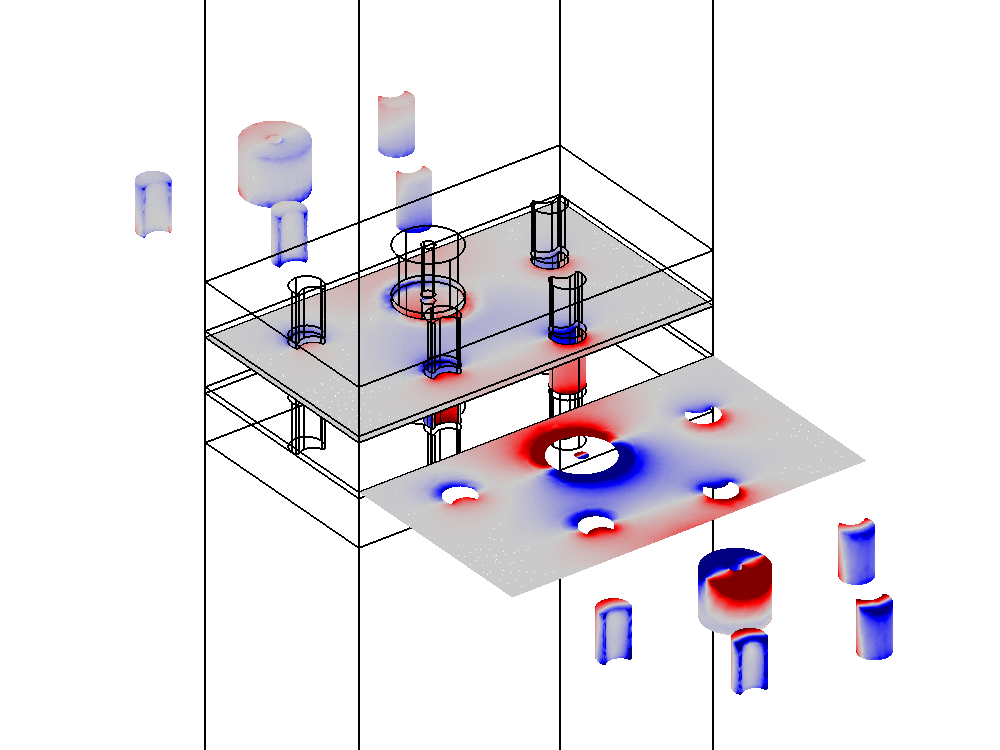

Supplement: Supplementary file 1 — Supplementary Information. [file 41598_2023_31685_MOESM1_ESM.zip › SREP-23-00542-s7.gif]

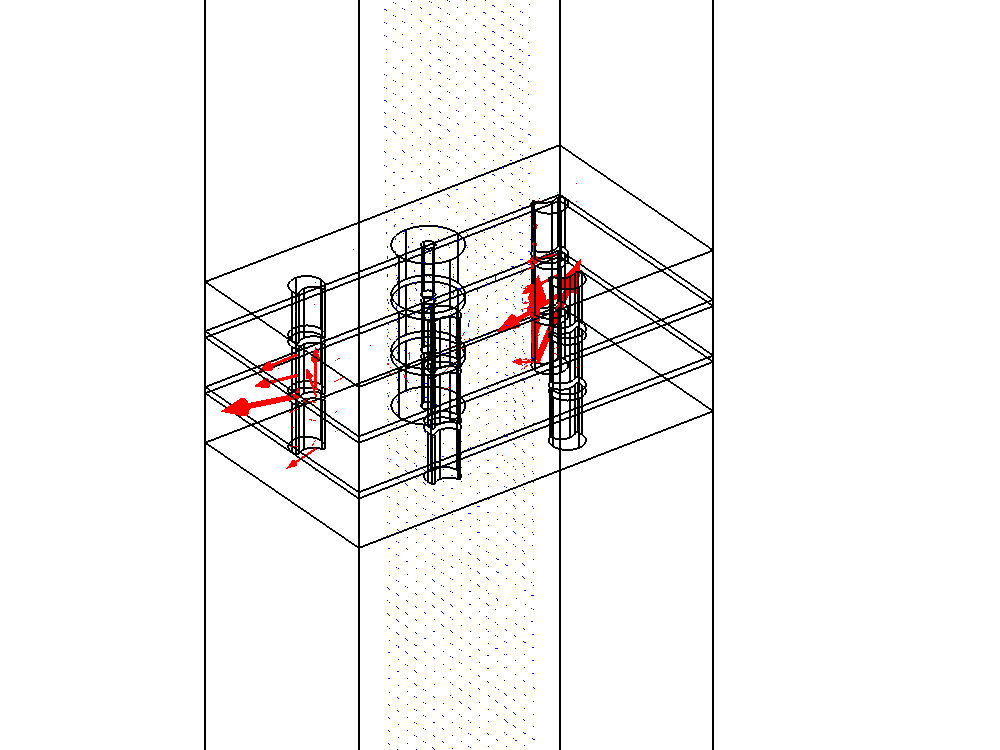

Supplement: Supplementary file 1 — Supplementary Information. [file 41598_2023_31685_MOESM1_ESM.zip › SREP-23-00542-s8.gif]

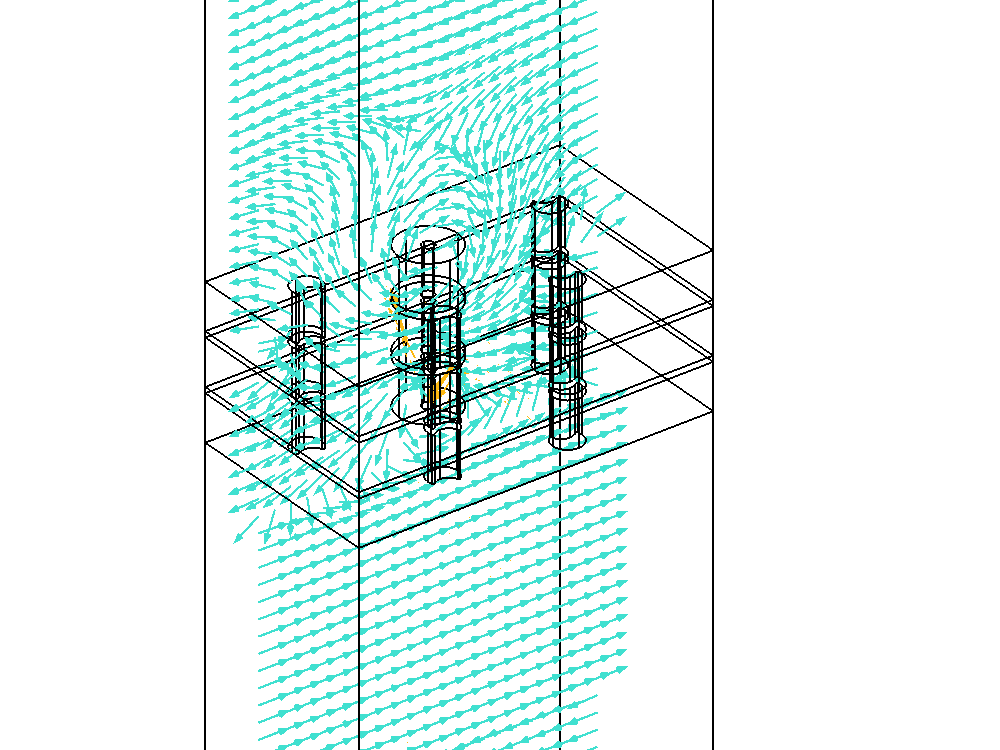

Supplement: Supplementary file 1 — Supplementary Information. [file 41598_2023_31685_MOESM1_ESM.zip › SREP-23-00542-s9.gif]
